# Supplementary material for: Red edge effect and chromoselective photocatalysis with amorphous covalent triazine-based frameworks
Source: Nat Commun. 2022 Apr 21;13:2171. doi: 10.1038/s41467-022-29781-9 (PMC9023581; doi:10.1038/s41467-022-29781-9)
Supplement: Supplementary file 1 — Supplementary Information [file 41467_2022_29781_MOESM1_ESM.pdf]

Supplementary Information for
Red Edge Effect and Chromoselective Photocatalysis with Amorphous Covalent Triazine-based Frameworks

Yajun Zou,[1,2] Sara Abednatanzi,[3] Parviz Gohari Derakhshandeh,[3] Stefano Mazzanti,[1] Christoph M. Schüßlbauer,[4] Daniel Cruz,[5,6] Pascal Van Der Voort,[3] Jian-Wen Shi,[2] Markus Antonietti,[1] Dirk M. Guldi,[4] Aleksandr Savateev[1,*]

1 Department of Colloid Chemistry, Max Planck Institute of Colloids and Interfaces, Am Mühlenberg 1, 14476 Potsdam, Germany

2 State Key Laboratory of Electrical Insulation and Power Equipment, Center of Nanomaterials for Renewable Energy, School of Electrical Engineering, Xi'an Jiaotong University, Xi'an 710049, P. R. China

3 Center for Ordered Materials, Organometallics and Catalysis, Ghent University, Krijgslaan 281-S3, 9000 Gent, Belgium

4 Department of Chemistry and Pharmacy Interdisciplinary Center for Molecular Materials (ICMM), Friedrich-Alexander University Erlangen-Nürnberg, Egerlandstraße 3, 91058 Erlangen, Germany

5 Department of Inorganic Chemistry, Fritz-Haber-Institut der Max-Planck-Gesellschaft, Berlin 14195,Germany

6 Department of Heterogeneous Reactions, Max Planck Institute for Chemical Energy Conversion, Mülheiman der Ruhr 45470, Germany

Contents

Supplementary Methods......6

Chemicals...... 6
Synthesis of CTFs...... 6
Light source...... 6
Powder X-ray diffraction (PXRD)...... 7
Fourier-transformed infrared (FT-IR)...... 7
XPS...... 7
Elemental analysis...... 7
Scanning electron microscopy (SEM) and energy-dispersive X-ray (EDX)...... 7
Transmission electron microscopy (TEM)...... 8
Nitrogen adsorption/desorption...... 8
Thermal gravimetric analysis (TGA)...... 8
Optical absorbance...... 8
Photoluminescence (PL) spectra...... 8
Time-resolved (TR) PL spectra...... 8
Nuclear magnetic resonance (NMR)...... 9
Mott-Schottky measurement...... 9
Electron paramagnetic resonance (EPR) study...... 10
EPR spectra measurement of CTFs and PYT/anisole mixture...... 10
DMPO-$O_2^{\bullet-}$ adduct detection...... 10
TEMPO detection...... 10
Low-temperature EPR spectra measurement...... 11
Ultraviolet photoelectron spectroscopy...... 11
Ultrafast pump-probe transient absorption spectroscopy (TAS)...... 11
Method of photocatalytic oxidative bromination of aromatic compounds (0.6 mmol scale reaction)...... 12
Method of photocatalytic oxidative bromination of aromatic compounds (6 mmol scale reaction)...... 12
Photocatalytic oxidative bromination of anisole with quenchers...... 12
Apparent quantum yield (AQY) measurement...... 13
Dual Ni-photocatalytic C-N cross-coupling...... 13
Amplitude average lifetime...... 13
Intensity average lifetime...... 14
AQY...... 14

Calculation of product yield and conversion of reagent in photocatalytic experiments 14

Supplementary Notes 15

Supplementary Note 1 15

Supplementary Note 2 16

Supplementary Note 3 16

Supplementary Note 4 17

Supplementary Note 5 17

Supplementary Note 6 18

Supplementary Note 7 19

Supplementary Note 8 19

Supplementary Figures 20

Supplementary Figure 1. Ideal chemical structures of CTFs. a) PHT. b) PYT. 20

Supplemenary Figure 2. Powder XRD patterns of CTFs. 21

Supplementary Figure 3. Electron microscopy images of CTFs. 22

Supplementary Figure 4. Transmission electron microscopy images of CTFs. 23

Supplementary Figure 5. Pore size distribution in CTFs. 24

Supplementary Figure 6. TGA curves of CTFs. 25

Supplementary Figure 7. Spectroscopic characterization of CTFs. 26

Supplementary Figure 8. Ultraviolet photoelectron spectra (UPS) of CTFs. 27

Supplementary Figure 9. Mott-Schottky plots. 28

Supplementary Figure 10. Synthesis and ideal structure of PYTnc. 29

Supplementary Figure 11. PL spectra of PHT and PYT upon excitation at 450 nm. 30

Supplementary Figure 12. 2D time-resolved (TR)-PL spectra of the CTFs upon excitation at λ_{exc} = 375, 470 and 640 nm with emission in the range λ_{em} = 400-900 nm. 31

Supplementary Figure 13. Example of the PL decay curves with instrument response function (IRF). 32

Supplementary Figure 14. Low-temperature EPR spectra of CTFs. a) PHT in dark. b) PYT in dark. Data were fitted. The g-factor of each EPR spectrum is given on the graph. 33

Supplementary Figure 15. Specific concentration of spins in PHT and PYT over irradiation time. 34

Supplementary Figure 16. Correlation between REE and E_g in CTFs. 35

Supplementary Figure 17. FT-IR spectra of fresh PYT and PYT recovered after photocatalytic bromination of anisole. 36

Supplementary Figure 18. Optical absorbance spectra of fresh PYT and PYT recovered after photocatalytic bromination of anisole. 37

Supplementary Figure 19. PL spectra of PYT recovered after the photocatalytic bromination of anisole upon a range of λ_{exc}. 38

Supplementary Figure 20. 2D time-resolved (TR)-PL spectra of PYT recovered after photocatalytic bromination of anisole excited at λ_{exc} = 375, 470 and 640 nm with emission at λ_{em} = 400-900 nm. 39

Supplementary Figure 21. AQY of photocatalytic bromination of anisole with the CTFs and the corresponding optical absorbance spectra. 40

Supplementary Figure 22. An exemplary ^{1}H NMR spectrum of anisole bromination reaction mixture. 41

Supplementary Figure 23. An image of the setup used for oxidative bromination performed on 6 mmol scale of anisole. 42

Supplementary Figure 24. ^{1}H NMR spectrum of the reaction mixture with anisole as substrate. . 43

Supplementary Figure 25. ^{1}H NMR spectrum of the reaction mixture with 4-bromo-2-chloroanisole as substrate. 44

Supplementary Figure 26. ^{1}H NMR spectrum of the reaction mixture with N,N-dimethylaniline as substrate. 45

Supplementary Figure 27. ^{1}H NMR spectrum of the reaction mixture with 1,3-dimethoxybenzene as substrate. 46

Supplementary Figure 28. ^{1}H NMR spectrum of the reaction mixture with 1,2,3-trimethoxybenzene as substrate. 47

Supplementary Figure 29. ^{1}H NMR spectrum of the reaction mixture with 1,3,5-trimethoxybenzene as substrate. 48

Supplementary Figure 30. Potentials of VB and CB in PHT and PYT and redox potentials of organic molecules. 49

Supplementary Figure 31. A summary of a mechanism of photocatalytic oxidative bromination that involves π–π^* and n–π^* excited states of CTFs. 50

Supplementary Figure 32. A list of arylbromides that did not give the C–N coupling products. .. 51

Supplementary Figure 33. Amplitude average lifetime of PYTnc obtained with λ_{exc} = 375, 470 and 640 nm and λ_{em} = 400-900 nm. 52

Supplementary Figure 34. Correlation between the surface area and cumulative pore volume of the CTFs, yield of 4-bromoanisole **2a** and the product of C–N cross-coupling **5a**. 53

Supplementary Figure 35. A tentative mechanism of Ni-dual photocatalytic C–N cross coupling mediated by PHT. 54

Supplementary Figure 36. PL spectra of PYTnc acquired at λ_{exc} 350, 365, 400, 450, 500, 550, 600 and 650 nm. 55

Supplementary Figure 37. Transient absorption spectroscopy of PYT. 56

Supplementary Figure 38. Transient absorption spectroscopy of PYTnc. 57

Supplementary Figure 39. Transient absorption spectroscopy of PHT. 58

Supplementary Figure 40. Schematic mechanism of anisole oxidative bromination with PYT. ... 59

Supplementary Figure 41. Positions of band edges in semiconductors and redox potentials of molecular sensitizers. 60

Supplementary Figure 42. Gas chromatogram of the reaction mixture and mass spectra of components using **3a** as the reagent. 61

Supplementary Figure 43. Gas chromatogram of the reaction mixture and mass spectra of components using **3b** as the reagent. 62

Supplementary Figure 44. Gas chromatogram of the reaction mixture and mass spectra of components using **3c** as the reagent. 63

Supplementary Figure 45. Gas chromatogram of the reaction mixture and mass spectra of components using **3d** as the reagent. 64

Supplementary Tables 67

Supplementary Table 1. Integrated area ratios of deconvoluted peaks in XPS spectra 67

Supplementary Table 2. Theoretical and measured elemental compositions of PHT and PYT. 68

Supplementary Table 3. Fluorescence lifetime of PHT and PYT. 69

Supplementary Table 4. Influence of explicitly added quantities of H_2O_2 on the yield of 4-bromoanisole. 76

Supplementary Table 5. Combustion elemental analysis of fresh PYT and recovered after the photocatalytic oxidative bromination of anisole. 76

Supplementary Table 6. Control experiments for leaching of photocatalytically active organic moieties from PYT. 77

Supplementary Table 7. Photocatalytic oxidative bromination with PYT versus oxidative bromination of electron rich aromatic compounds using a mixture of H_2O_2 and HBr in dark. 78

Supplementary Table 8. Oxidative photocatalytic bromination of 1,3,5-trimethoxybenzene with KBr. 80

Supplementary Table 9. Photocatalytic oxidative bromination of 3,4-ethylenedioxythiophene with PYT using KBr as bromine source. 81

Supplementary Table 10. Role of acid in photocatalytic oxidative halogenation of electron rich aromatic compounds. 82

Supplementary Table 11. An attempt to enable photocatalytic oxidative bromination of anisole with PYT using KBr as bromine source. 83

Supplementary Table 12. Combustion elemental analysis of fresh PHT and recovered after dual Ni-photocatalytic C–N cross-coupling. 83

Supplementary References 84

Supplementary Methods

Chemicals

Anisole (≥99%) was purchased from Fluka. 2-Chloroanisle (>97%) was purchased from TCL. N,N-dimethylaniline (99%) was purchased from Acros Organics. 1,3-Dimethoxybenzene (99%) was purchased from Acros Organics. 1,2,3-trimethoxybenzene (>99%) was purchased from TCI. 1,3,5-trimethoxybenzene (>98%) was purchased from TCI. 1,4-Dinitrobenzene (>98%) was purchased from Alfa Aesar. Acetonitrile (hypergrade for LC-MC) was purchased from Merck. HBr (aqueous, 48 wt. %) was purchased from Sigma Aldrich. 5,5-Dimethyl-1-pyrroline-N-oxide (DMPO) (≥98%) was purchased from Enzo. 2,2,6,6-Tetramethylpiperidine (TEMP) (>98%) was purchased from Alfa Aesar. Benzoquinone (≥98%) was purchased from Sigma-Aldrich. Sodium azide (≥ 99.5%) was purchased from Sigma-Aldrich. Deuterated chloroform ($CDCl_3$) (99.8 atom%) was purchased from Sigma-Aldrich. Nafion D-520 dispersion (5 wt. % in mixture of lower aliphatic alcohols and water, contains 45% water) was purchased from Sigma-Aldrich. Na_2SO_4 (anhydrous, 99.0-100.5%) was purchased from Sigma-Aldrich. Fluorine doped tin oxide (FTO) coated glass was purchased from Sigma-Aldrich. Capillary (IntraMark, volume 50 μL) was purchased from BRAND GMBH + CO KG.

Synthesis of CTFs

PYTnc. Terephthalamidine dihydrochloride (235.5 mg, 1 mmol), 2,6-bis[(phenylimino)methyl]pyridine (142.6 mg, 0.5 mmol), and cesium carbonate (716 mg, 2.2 mmol) were added to a solution of DMSO (15.0 mL) in 50 mL round-bottom flask. The mixture was heated to 100 °C for 24 h and then the temperature of the reaction was raised to 150 °C for 36 h. After cooling down to room temperature, the resulting precipitate was washed with diluted HCl (3 × 20 mL) to remove the salt and residual cesium carbonate followed by washing with water (3 × 30 mL), acetone (3 × 10 mL), and THF (3 × 10 mL), before drying at 80 °C under vacuum for 12 h. Finally, PYTnc was obtained as a yellow powder.

Light source

In this work the following light sources were used: **blue LED module 1** (home-made steel cylinder photoreactor attached with self-adhesive LED strips which were purchased from JKL components, emission maximum λ = 468 nm, measured optical power 14 mW cm^{-2} at the central position); **blue LED module 2** (emission maximum λ = 461 nm, measured optical

power 101 mW cm^{-2}); **blue LED module 3** (M455F3, purchased from ThorLabs) (emission maximum λ = 455 nm) coupled with an optical fiber and controlled by the driver (DC2200, purchased from ThorLabs); **green LED module** (M530F2, purchased from ThorLabs) (emission maximum λ = 530 nm, measured optical power 41 mW cm^{-2}) coupled with an optical fiber and controlled by the driver (DC2200, purchased from ThorLabs); **red LED module** (emission maximum λ = 620-625 nm, measured optical power 302 mW cm^{-2}); **white LED module** (emission maximum λ = 400-760 nm, measured optical power 203 mW cm^{-2}). Irradiance of LED modules was measured using PM400 Optical Power and Energy Meter equipped with the integrating sphere S142C and purchased from Thorlabs.

Powder X-ray diffraction (PXRD)

PXRD patterns were recorded at room temperature on a Bruker D8 X-Ray Diffractometer using Cu Kα1 radiation.

Fourier-transformed infrared (FT-IR)

FT-IR spectra were recorded on Thermo Scientific Nicolet iD5 spectrometer equipped with an attenuated total reflection unit applying a resolution of 2 cm^{-1}.

XPS

XPS analysis was carried out on a Thermo Fisher Scientific ESCALAB spectrometer with Al Kα radiation.

Elemental analysis

Elemental analysis was accomplished by combustion analysis using a Vario Micro device.

Scanning electron microscopy (SEM) and energy-dispersive X-ray (EDX)

SEM images were obtained on JSM-7500F (JEOL) at an accelerating voltage of 3 kV. EDX) investigations were conducted using a Link ISIS-300 system (Oxford Microanalysis Group) equipped with a Si(Li) detector and an energy resolution of 133 eV.

Transmission electron microscopy (TEM)

The TEM study was performed using a double Cs corrected JEOL JEM-ARM200F (S)TEM operated at 80 kV equipped with a cold field emission gun.

Nitrogen adsorption/desorption

Nitrogen adsorption/desorption measurements were performed after degassing the samples at 150 °C for 20 hours using a Quantachrome Quadrasorb SI-MP porosimeter at 77 K. The specific surface areas were calculated by applying the Brunauer-Emmett-Teller (BET) model to adsorption isotherms for $0.05<p/p_0<0.3$ using the QuadraWin 5.05 software package. The pore size distribution was obtained by applying the quenched solid density functional theory (QSDFT) model for N_2 adsorbed on carbon with cylindrical pore shape at 77 K.

Thermal gravimetric analysis (TGA)

TGA measurement was performed using a thermo microbalance TG 209 F1 Libra coupled with a Thermostar Mass spectrometer (Pfeiffer Vacuum) with an ionization energy of 75 eV. Analysis was conducted under N_2 atmosphere.

Optical absorbance

Optical absorbance spectra were measured on Shimadzu UV 2600 spectrophotometer equipped with an integrating sphere.

Photoluminescence (PL) spectra

PL spectra were recorded on FP-8300 fluorescence spectrometer.

Time-resolved (TR) PL spectra

TR-PL spectra were recorded on fluorescence lifetime spectrometer (Fluo Time 250, PicoQuant) equipped with PDL 800-D picosecond pulsed diode laser driver. The decay curves were fitted using a nonlinear method with a multicomponent decay law given by

$$I\,(t) = a_1 \exp(-t/\tau_1) + a_2 \exp(-t/\tau_2) + a_1 \exp(-t/\tau_3) \qquad (1)$$

The solid-state TR PL spectra were obtained with λ_{exc} = 375, 470 and 640 nm, respectively. The following settings were used for the spectra acquisition: Laser Frequency 40 MHz, Emission Monochromator Bandwidth 2 nm, Delta (step between λ_{em}) 10 nm. Long pass filters of 495 and 665 nm were used for λ_{exc} = 470 and 640 nm, respectively.

For the quenching experiment, sample suspension containing 8 mg of PYT in 20 mL MeCN/H_2O (volume ratio 5:1) solvent was prepared and sonicated for 1 h before use. The TR PL spectra were obtained with λ_{exc} = 470 nm and λ_{em} = 580 nm. The following settings were used for the spectra acquisition: Laser Frequency 40 MHz, Emission Monochromator Bandwidth 10 nm.

Nuclear magnetic resonance (NMR)

^{1}H NMR spectra were recorded on Agilent 400 MHz. Chemical shifts are reported in ppm versus solvent residual peak: chloroform-d 7.26 ppm.

Mott-Schottky measurement

The Mott-Schottky measurement was carried out with Arbin electrochemical testing station (Arbin Instrument) in a standard three-electrode quartz cell. The working electrode was prepared as follows: 2 mg of sample was suspended in 0.2 mL of deionized water containing 0.02 mL of 5 wt% Nafion D-520 dispersion, and the mixture was then dispersed by ultrasonication and spread onto an FTO glass. After being dried naturally, the FTO glass was heated at 120 °C for 1 h. The prepared thin film was employed as working electrode, with a platinum plate as counter electrode and Ag/AgCl as reference electrode (3 M KCl). 0.5 M Na_2SO_4 aqueous solution was used as electrolyte. The measurement was carried out upon a frequency of 10 kHz in a potential range from –1.0 to 0.4 V vs. Ag/AgCl. The measured potentials vs. Ag/AgCl were converted to the reversible hydrogen electrode (RHE) scale according to the Nernst equation:

$$E_{\mathrm{RHE}} = E_{\mathrm{Ag/AgCl}} + E^{0}_{\mathrm{Ag/AgCl}} + 0.059\ \mathrm{pH} \qquad (2)$$

where E_{RHE} is the converted potential vs. RHE, $E^{o}_{\mathrm{Ag/AgCl}}$ = 0.1976 at 25 °C, and $E_{\mathrm{Ag/AgCl}}$ is the experimentally measured potential against Ag/AgCl reference.

Electron paramagnetic resonance (EPR) study

EPR study was conducted on Bruker EMXnano benchtop X-Band EPR spectrometer. The following settings were used for the spectra acquisition unless other is specified: Center Field 3444.05 G, Sweep Width 100 G, Receiver Gain 40 dB, Modulation Amplitude 1.000 G, Number of Scans 16, Microwave Attenuation 25 dB. For '2D field delay experiment': Delay 60 s, Number of Points 16. Blue LED module 3 (455 nm, 155 mW cm^{-2}) coupled with an optical fiber was used as the light source for the dynamic measurement.

EPR spectra measurement of CTFs and PYT/anisole mixture

Two capillaries were sealed from one side with the flame of gas burner. Each capillary was filled with PHT and PYT powder (3 mg). EPR spectra were acquired in dark. Dynamic EPR spectra under illumination were recorded using a '2D field delay' mode, with the first spectrum recorded when LED is OFF, the rest under illumination. The total illumination time was ~ 40 min.

Anisole (20 μL) was then added to the PYT capillary which was subsequently centrifuged at 3000 rpm for 5 min to make sure the catalyst was completely immersed in anisole. Acquisition of EPR in dark and dynamic EPR under light irradiation was repeated.

DMPO-$O_2^{\bullet -}$ adduct detection

PYT (3 mg) and 40 mM DMPO in methanol (40 μL) were added to the capillary. The capillary was centrifuged at 3000 rpm for 5 min. EPR spectra in dark and dynamic EPR spectra under illumination were acquired (~ 40 min).

TEMPO detection

A mixture of PYT (5 mg) and TEMP (8.5 μL) in MeCN (3 mL) was added to a glass tube with an inlet for gas connection and a ground joint for 'cold finger' connection. The mixture was vigorously stirred at room temperature for 10 min. A capillary was charged with the reaction mixture (40 μL). EPR spectrum in dark was acquired. The reaction mixture was purged with O_2 for 30 s. A cold finger was immersed into the suspension and cooling water circulation was enabled maintaining the reaction mixture temperature at 20-25 ^{o}C. A balloon with O_2 was connected to the reactor headspace via gas inlet. The reaction mixture was vigorously stirred

under blue light irradiation (blue LED module 2, 101 mW cm^{-2}) for 3 h. A capillary was charged with the reaction mixture (40 μL) and EPR spectrum acquisition in dark was repeated.

Low-temperature EPR spectra measurement

Low-temperature EPR spectra were acquired with liquid nitrogen cooling which allowed stepwise decrease of temperature from 220 to 90 K. The following settings were used for the spectra acquisition: Center Field 3448.05 G, Sweep Width 200 G, Receiver Gain 50 dB, Modulation Amplitude 1.000 G, Number of Scans 16, Microwave Attenuation 15 dB (equivalent to 0.03162 mW microwave power).

Ultraviolet photoelectron spectroscopy

The ultraviolet photoelectron spectroscopy (UPS) spectra were acquired with a He I (21.2 eV) radiation source. The detector was a combined lens with an analyzer module thermoVG (TLAM). VB potential (U, V vs SHE, electrochemical scale) of the materials was calculated using the UPS data (physical scale) according to the equation:

$$U = k \cdot (E - 4.44) \tag{3}$$

where E – energy of the VB determined from the UPS, eV; k – conversion factor, 1 V eV^{-1}.

Ultrafast pump-probe transient absorption spectroscopy (TAS)

Investigation was performed using a Clark MXR CPA 2101 Ti:sapphire as the laser source (775 nm, 1 kHz, 150 fs pulse width). Time-resolved transient absorption spectra were acquired on a sub-ps and ns resolution, using an Ultrafast Systems HELIOS or EOS fs/ns transient absorption spectrometer with time delays from 0 to 5500 ps and 1 ns to 375 μs, respectively. For sub-ps, white light for the probing pulse in the visible region of the optical spectrum (~450-750 nm) was generated by focusing part of the fundamental 775 nm output onto a 2 mm sapphire disk. For (near) IR (800-1350 nm) white light, a 10 mm sapphire was used. For ns timescale experiments, white light for probing was generated by a photonic crystal fiber supercontinuum laser with a 1064 nm fundamental. The excitation wavelength was generated via the second harmonic of the fundamental CPA laser wavelength and the energy per pulse reduced to 2 μJ using neutral density filter. For all TAS measurements, 5 wt%. dispersions of

the respective polymer in MeCN were used. The samples were sonicated for approximately 10 h before conducting measurements.

Method of photocatalytic oxidative bromination of aromatic compounds (0.6 mmol scale reaction)

A glass tube (5 mL) was charged with a mixture of substrate (600 μmol), HBr (0.6 mL, 48 wt. %), PYT (4 mg) and acetonitrile (3 mL). Magnetic stir bar was placed in the tube. The tube was closed with the rubber septum and a ballon filled with O_2 was connected to the reaction mixture head space via needle. The reaction mixture was vigrously stirred under blue LED module 2 (101 mW cm^{-2}) for entry 1 and 3-5 and white LED module for entry 2 (203 mW cm^{-2}) (Table 2). The reaction progress was checked with thin layer chromotography every 24 h. After the substrate was completely consumed, the product was extracted with $CHCl_3$, dried over anhydrous Na_2SO_4, concentrated in vacuum (50 °C, 30 mbar) and measured with ^1H NMR without internal standard.

Method of photocatalytic oxidative bromination of aromatic compounds (6 mmol scale reaction)

A 3 neck custom made flask (100 mL) was charged with a mixture of anisole (652 µL, 6 mmol), HBr (6 mL, 48 wt. %), PYT (40 mg) and acetonitrile (30 mL). Magnetic stir bar was placed in the flask. Then, a cold finger attached to water cooling was immersed into the photoreactor. Temperature of the reaction mixture was maintained at 20-25 °C enabling the circulation water. The ballon filled with O_2 was attached to one of the necks. The pressure of O_2 during the experiment was maintained at ca. 1 bar. The reaction mixture was vigrously stirred for 48 h under illumination with 3 LED modules (emission maximum λ = 465 nm) with the distance between the photoreactor and LEDs of 1 cm (optical power supplied to the photoreactor by 3 LEDs was 1110 mW·cm^{-2}). Yield and conversion were determined by ^1H NMR with internal standard.

Photocatalytic oxidative bromination of anisole with quenchers

A glass vial (4 mL) was charged with a mixture of anisole (2.2 µL, 20 µmol), HBr (0.1 mL, 48 wt. %), photocatalyst (4 mg) and acetonitrile (0.5 mL). Benzoquinone (1 mg) or sodium azide (0.65 mg) was added separately to quench $O_2^{\bullet-}$ and $^1O_2$, respectively. Magnetic stirring bar was placed in the vial. The vial was purged with O_2 for 30 s and closed with cap. The

reaction mixture was vigrously stirred in the photoreactor under illumination with blue LED module 1 (14 mW cm^{-2}) for 4 h. After the reaction $CDCl_3$ (0.7 mL) and water (0.1 mL) were added to the reaction mixture. The organic layer was separated and measured with ^{1}H NMR using 1,4-dinitrobenzene as internal standard.

Apparent quantum yield (AQY) measurement

A glass tube (5 mL) was charged with a mixture of anisole (600 µmol), HBr (0.6 mL, 48 wt. %), photocatalyst (4 mg) and acetonitrile (3 mL). Magnetic stir bar was placed in the tube. The tube was closed with the rubber septum and a ballon filled with O_2 was connected to the reaction mixture head space via needle. The reaction mixture was vigrously stirred under blue LED module 2 (455 nm, 47 mW cm^{-2}) or green LED module (530 nm, 41 mW cm^{-2}), respectively. After 24 h, the product was extracted with $CHCl_3$, dried over anhydrous Na_2SO_4, concentrated in vacuum (50 °C, 30 mbar) and measured with ^{1}H NMR with 1,4-dinitrobenzene as internal standard.

Dual Ni-photocatalytic C-N cross-coupling

A glass tube (5 mL) was charged with a magnetic stirring bar, aryl halide (0.05 mmol, 1.0 equiv), PHT (12.0 mg), $NiBr_2$·glyme (0.0025 mmol, 0.05 equiv, 0.8 mg), pyrrolidine (0.09 mmol, 1.8 equiv, 7.4 µL), and DABCO (0.11 mmol, 2.2 equiv, 12.3 mg). Then, 1.0 mL of dimethylacetamide (DMA) was added. The reaction mixture was purged with nitrogen atmosphere via double needle technique for 2 minutes and closed. The reaction was vigorously stirred under red LED module (λ = 620-625 nm, 302 mW·cm^{-2}) for 168 hours. After, the reaction mixture was centrifuged to remove the solid catalyst and then analyzed. Yield and conversion have been determined via GC–MS analysis.

Amplitude average lifetime

$$\bar{\tau} = \frac{\sum a_i \tau_i}{\sum a_i} \qquad (4)$$

where $\bar{\tau}$ is the average fluoresence lifetime, a_i is the amplitude fraction and τ_i is the fluorescence lifetime.

Intensity average lifetime

$$\bar{\tau} = \frac{\sum a_i \tau_i^2}{\sum a_i \tau_i} \tag{5}$$

where $\bar{\tau}$ is the average fluoresence lifetime, a_i is the amplitude fraction and τ_i is the fluorescence lifetime.

AQY

The AQY was calculated as:

$$AQY = \frac{n_{product}}{n_{photon}} \times 100\ \% = \frac{w \times N_A \times \varepsilon}{I \times S \times t \times \frac{\lambda}{hc}} \times 100\ \% \tag{6}$$

where AQY – the apparent quantum yield; $n_{product}$ – the number of product molecules; n_{photon} – the number of incident photons; w – the mole of product, mol; N_A – the Avogadro constant, 6.02×10^{23} mol^{-1}; ε – the number of electrons transferred between catalyst and a reactant molecule; I – the light intensity, W cm^{-2}; S – the irradiation area, cm^2; t – the duration of the experiment, s; λ – the wavelength of incident light, m; h – the Planck's constant, 6.626×10^{-34} J s; c – the speed of light, 3×10^8 m s^{-1}.

Calculation of product yield and conversion of reagent in photocatalytic experiments

Yield of a product was calculated according to the equation:

$$Yield = \frac{n_{exp}}{n_{theor}} 100\% \tag{7}$$

where n_{exp} – amount of a product formed in a photocatalytic experiment and determined using either ^{1}H NMR or GC-MS, mol; n_{theor} – amount of a product expected to form in a photocatalytic experiment according to the reaction stoichiometry, mol.

Conversion of a reagent was calculated according to the equation:

$$Conversion = \left(1 - \frac{n_{rxn}}{n_{load}}\right) 100\% \tag{8}$$

Where n_{rxn} – amount of a reagent remaining in the reaction mixture after a photocatalytic experiment and determined using either ^{1}H NMR or GC-MS, mol; n_{load} – amount of a reagent taken for a photocatalytic experiment, mol.

Supplementary Notes

Supplementary Note 1

A more crystalline material possessing pyridine-2,6-diyl linkers (PYTnc) was synthesized by polycondensation of 2,6-bis[(phenylimino)methyl]pyridine with terephthalamidine dihydrochloride (Supplementary Figure 10). Using the Schiff base instead of pyridine-2,6-dicarboxaldehyde, polycondensation is expected to proceed slower and as a result would yield more ordered material. Improved crystallinity of PYTnc compared to PYT is supported by more pronounced diffraction peak at 18.05 degrees in the PXRD (Supplementary Figure 2). Still, PYTnc is nanocrystalline material with the crystallites diameter estimated from the Scherrer equation to be ca. 3 nm.

$$\tau = \frac{K \cdot \lambda}{\beta \cdot cos\theta} \quad (9)$$

where τ – the mean size of the crystallite, nm; K – dimensionless shape factor, assumed to be 0.9; λ – wavelength of the diffractometer, nm; β – broadening (FWHM) of the diffraction line, degree; θ – Bragg angle, degree.

PYTc was characterized by a set of spectroscopic techniques, similar to PYT and PHT. In the DRUV-vis absorption spectrum (Supplementary Figure 7a), PYTnc shows absorption edge at ca. 600 nm. Using this absorption edge, the optical gap defined by π–π* transitions was calculated to be 1.92 eV (Supplementary Figure 7b). As indicated by the absorption features stretching up to nIR, PYTnc also contains intraband states.

Upon excitation at λ_{exc} 350 nm, PYTnc emits photons with the maximum at 650 nm (Supplementary Figure 36). Compared to PYT, the emission peak is red-shifted due to narrower optical gap. Similar to PHT and PYT, Stokes shift in PYTnc is ca. 50 nm at λ_{exc} 350 nm. When λ_{exc} exceeds ca. 550 nm, the emission peaks starts progressively shifting to longer wavelength – PYTnc also shows REE. The optical gap defined by π–π* transitions correlates with the onset of REE in the studied materials (Supplementary Figure 16a,b).

Unlike to PHT and PYT, in TR-PL spectra, PYTnc shows non-monotonous dependence of the amplitude average lifetime ($\bar{\tau}$, Supplementary Figure 33). Thus, upon excitation at 375 nm the maximum amplitude average lifetime is 0.330 ns, followed by 0.490 ns (upon excitation at 470 nm) and 0.087 ns (upon excitation at 640 nm).

Supplementary Note 2

Excitons of different energy are separated within different time. For example, as shown in Figure 3e (curve for PYT upon excitation at 470 nm), excitons with the energy 2.36 eV (corresponds to the wavelength 525 nm) are separated within 0.523 ns, those with the energy ca. 1.92 eV (645 nm) – within 0.607 ns and excitons with the energy 1.43 eV (865 nm) – within 0.28 ns. On the other hand, upon excitation of PYT at 640 nm, dependence of exciton energy and the time of their separation is a quasi linear – excitons with the energy 1.78 eV (695 nm) are separated within 0.981 ns, while those with the energy 1.39 eV (895 nm) – within 0.322 ns.

Supplementary Note 3

To further investigate the excited state behavior and get a closer look at the quantum states of the photo-excited charge carriers involved, we performed ultrafast pump-probe transient absorption measurements. For PYT (Supplementary Figure 37), directly after photoexcitation at 387 nm (2 μJ) negative differential absorptions form from the ground state bleaching (GSB) up to 900 nm together with the formation of positive differential absorptions in the nIR region commencing with wavelengths >900 nm up to 1350 nm (end of detectable range). Superimposed to the GSB are excited state absorptions maximizing at 510 which intensity slightly increase within the first 145 ps. The transient absorptions in the nIR region decay within the first 9 ns, whereas the maximum in the visible region take around 30 ns to transform into one broad negative signal minimizing at around 520 nm and reaching up to beyond >1350 nm. All signals then decay back to 0 within a timeframe of 250 μs.

We found that increasing the crystallinity has a strong effect on the excited state dynamics of PYT. For the nanocrystalline form of PYT, PYTnc, photoexcitation leads to the immediate formation of broad excited state absorptions (ESA) maximizing at 495, 560, and 1300 nm (Supplementary Figure 38). The ESA in the visible region quickly decay within the first 100 ps giving rise to a negative signal which stems from the GSB. On the other hand, the positive differential absorptions in the nIR decay back to zero within 4 ns. The GSB then commences to return to zero within 75 μs. The faster transformation of all components in nanocrystalline PYT corroborates the importance and influence of the structure and crystallinity of the photo-catalyst. A possible rationale for the overall faster transformation could be that less trap states are present with higher crystallinity that slow down or even hinder certain deactivation pathways that could be beneficial for the photocatalytic conversions. Another possibility, which goes hand in hand with the decrease of fluorescence lifetime and higher catalytic activity

observed in oxidative halogenation of anisole (Table 1), is that increase in crystallinity may be associated with an easier charge transfer to possible surface states.

Moving to dispersions of PHT (Supplementary Figure 39), we find significantly weaker signals as well as strong scattering upon photoexcitation at 387 nm as compared to PYT and PYTnc. This likely correlates to the smaller size of the particles. Nonetheless, directly after photoexcitation with 387 nm, we find a negative transient from <460 nm to approximately 1000 nm together with the formation of an ESA in the nIR maximizing at around 1300 nm. The latter decays to zero within 1.5 ns. Furthermore, it must be noted that after photoexcitation, an ESA becomes apparent superimposed to the GSB in the region between <460 and 620 nm. After approximately 75 ps, these ESA increase in intensity giving rise to a positive differential absorption which converts into a broad GSB within 20 ns. Commencing with delay times >250 μs, all differential absorptions stemming from the GSB have returned to zero.

Supplementary Note 4

Visible-light absorption of the CTF generates excited electron-hole pairs, followed by their separation into free charges (Supplementary Figure 31). In the electrophilic substitution pathway, the photoexcited electrons reduce O_2 to H_2O_2, which further reacts with HBr to produce the active electrophilic species, denoted collectively as "HOBr". Meanwhile, Br_2 produced by oxidation of Br^- reacts with H_2O to give "HOBr" species. The "HOBr" subsequently reacts with anisole via electrophilic substitution resulting in the intermediate cation species. Further liberation of a proton yields the bromination product. In another pathway of nucleophilic bromination, one-electron reduction leads to the $O_2^{\bullet-}$, while energy transfer from the triplet excited state CTF affords $^1O_2$. The $O_2^{\bullet-}$ subsequently abstracts a proton from HBr to give the highly reactive $\bullet O_2H$ radical. Meanwhile, anisole is oxidized by the photogenerated holes into a radical cation, which readily reacts with Br^- via nucleophilic attack giving rise to the brominated neutral radical intermediate. The oxidation of the intermediate to the ultimate product, 4-bromoanisole, can either take place with $\bullet O_2H$ to give H_2O_2 or with $^1O_2$ to give $\bullet O_2H$. Given the fact that H_2O_2 could be also a potential oxidant, the side products can then participate in the oxidation reaction.[1]

Supplementary Note 5

Analysis of our own results and results obtained by other research groups, point that in case of nitrogen-rich conjugated organic polymers, the role of proton in the photocatalytic mechanism is not only to participate in reduction of O_2 to H_2O_2 (or $HO_2^{\bullet}$) via PCET, protons in fact shift VB level to more positive values. Such conclusion is in agreement with the results obtained by

Wu et al. for protonated g-C_3N_4.[2] Therein, protonation shifts the VB potential by 0.77V from 1.6 V to 2.37 V vs NHE.[2] Stabilization by 0.77 V also pulls down the potential of the CB, so that optical gap remains nearly constant. Protons (and in general cations) shift the CB and VB levels in nanoparticles of inorganic semiconductors, such as ZnO, to more positive values.[3] Protonation of a photocatalyst surface increases oxidation power of the photogenerated holes compared to that under neutral pH.

Using structure of PYT as an example, schematically the mechanism of oxidative bromination of anisole is depicted in Supplementary Figure 40. Protonation of certain pyridine units in PYT gives $[H\text{-}PYT]^{+}Br^{-}$. Upon excitation, electron transfer from the VB, which is localized at *p*-phenylene moieties (the electron-enriched part of the polymer) to the CB, which is localized at protonated pyridine-2,6-diyl and/or triazine-linkers (the electron-deficient part of the polymer), gives excited state $[H\text{-}PYT]^{+*}Br^{-}$. One-electron oxidation of anisole followed by nucleophilic attack of bromide anion and abstraction of hydrogen atom by hydroperoxyl radical gives 4-bromoanisole and transient $[H\text{-}PYT]^{\bullet}$. Open-shell character of the tentative $[H\text{-}PYT]^{\bullet}$ intermediate has been partially confirmed by increase of the amplitude of the EPR signal upon illumination of the mixture of PYT and anisole (Figure 4a). The latter transfers hydrogen atom to oxygen, which closes the photocatalytic cycle.

The suggested mechanism can rationalize the fact that in acidic environment we obtained 4-bromoanisole from anisole, despite its oxidation potential +1.81 V vs SCE[4] is more positive that the VB potentials of PHT (+1.55 V vs SCE) and PYT (+0.75 V vs SCE). Therefore, in acidic environment the VB potential of PHT is shifted by at least 0.26 V to more positive values. As a result, oxidation of anisole by the photocatalyst becomes feasible. Taking into account the band diagram of PYT, such shift is even larger, ca. 1.06 V, but due to higher content of pyridinic-nitrogen atoms (basic sites where protons reside) and 4 times larger surface area, is apparently feasible.

Supplementary Note 6

To check stability of PHT, we recovered the CTF and characterized by a series of techniques. FT-IR revealed that chemical structure of PHT was not altered (Supplementary Figure 46). Steady-state photoluminescence spectra of PHT acquired under several excitation wavelengths (Supplementary Figure 47), revealed that red edge effect was preserved. Combustion C, N, H analysis of fresh PHT and PHT recovered after dual Ni-photocatalytic C–N cross-coupling of pyrrolidine and 4-bromobenzonitrile revealed nearly identical chemical composition

(Supplementary Table 12). Moreover, C/N ratio in fresh PHT (3.81) was close to that in PHT recovered after the photocatalytic experiment (3.86).

Supplementary Note 7

Stronger oxidation power of the PHT excited state, 1.55 V compared to 0.75 V vs SCE in PYT, is advantageous for one-electron oxidation of the Ni(II)-intermediate (Supplementary Figure 41). Indeed, VB levels in PHT, mpg-CN(reference [5]) and Ir[dF(CF)$_3$ppy]$_2$(dtbbpy)PF$_6$(reference [6]) are 0.45-0.75 V more positive than the VB level in PYT (Supplementary Figure 41). PHT, mpg-CN(reference [5]) and Ir[dF(CF)$_3$ppy]$_2$(dtbbpy)PF$_6$(reference [6]) gave the C–N coupling product, while PYT did not.

At the same time, moderately reducing power of PHT excited state, CB −1.17 V compared to −1.75V vs SCE in PYT, is sufficient to enable one-electron reduction of Ni(I) intermediate as the primary pathway for catalyst turnover.[6] The undesirable dehalogenation process, which typically requires strongly reductive photocatalyst excited state,[7,8,9] is mitigated.

Supplementary Note 8

Data shown in Figure 2e and Supplementary Figure 33 was used to determine the longest time of photogenerated charge carriers ($\bar{\tau}_{max}$) separation. For PHT $\bar{\tau}_{max}$ is 0.753 ns (at λ_{exc} = 470 nm) and 0.83 ns (at λ_{exc} = 640 nm); for PYT is 0.627 ns (at λ_{exc} = 470 nm) and 0.981 ns (at λ_{exc} = 640 nm); for PYTnc is 0.489 ns (at λ_{exc} = 470 nm) and 0.085 ns (at λ_{exc} = 640 nm).

Supplementary Figures

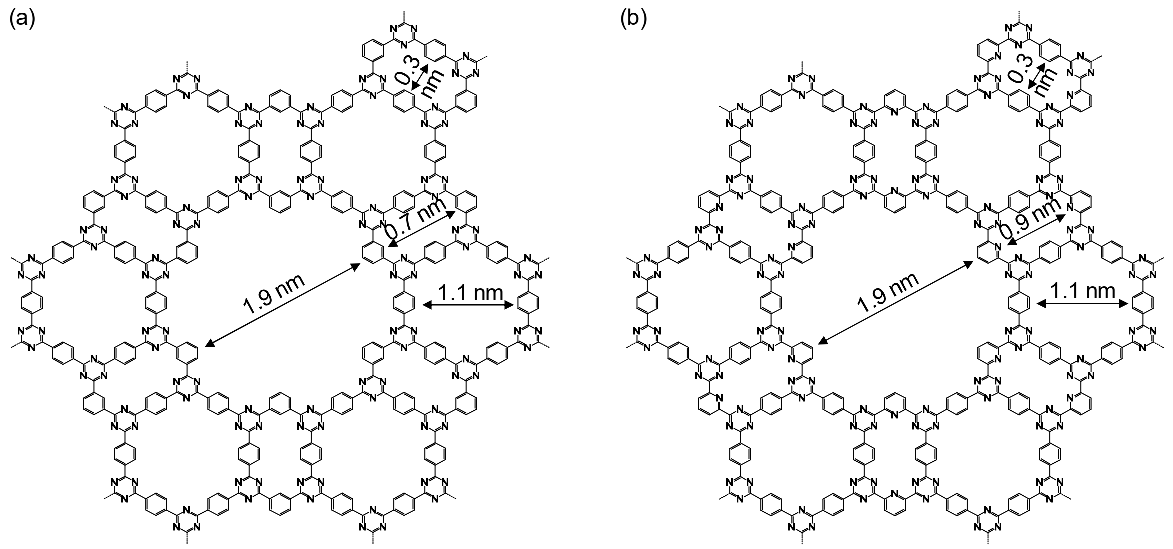

Supplementary Figure 1. Ideal chemical structures of CTFs. a) PHT. b) PYT.

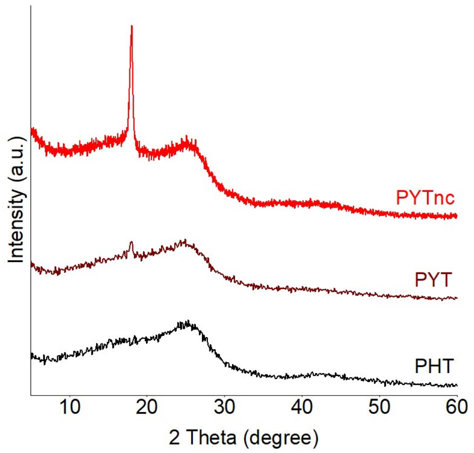

Supplemenary Figure 2. Powder XRD patterns of CTFs.

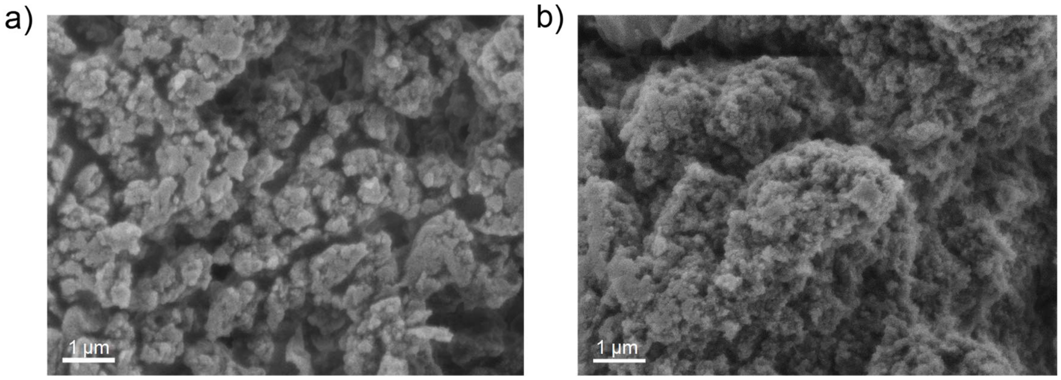

Supplementary Figure 3. Electron microscopy images of CTFs. a) SEM image of PHT. b) SEM image of PYT.

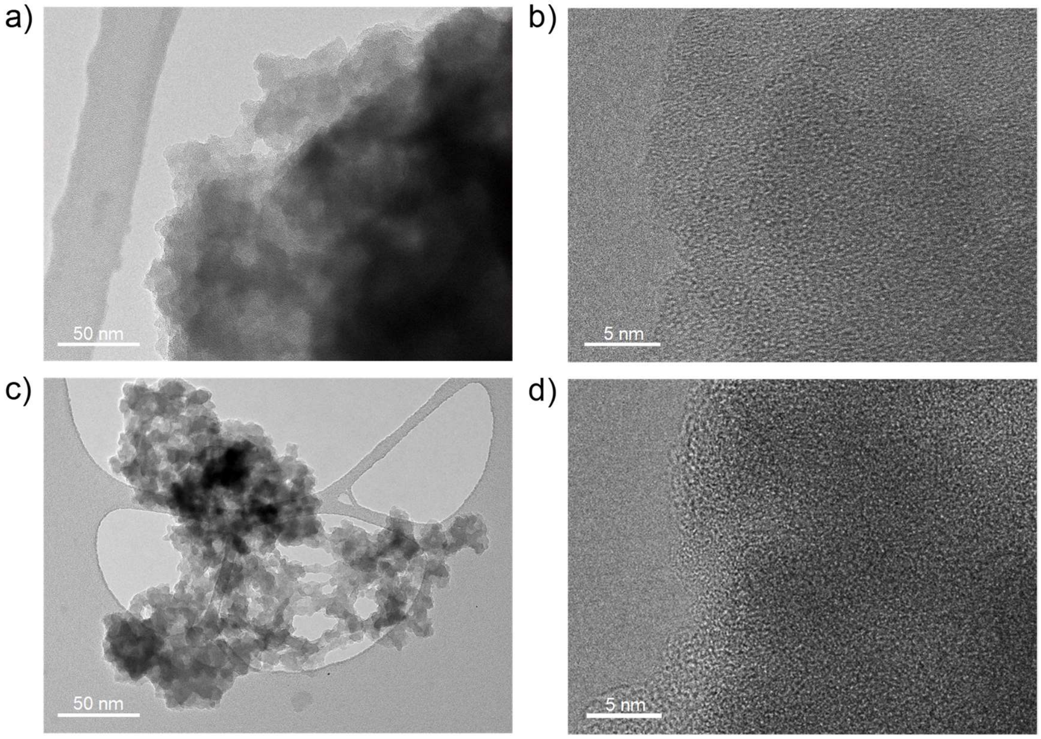

Supplementary Figure 4. Transmission electron microscopy images of CTFs. a, b) TEM images of PHT. c, d) TEM images of PYT.

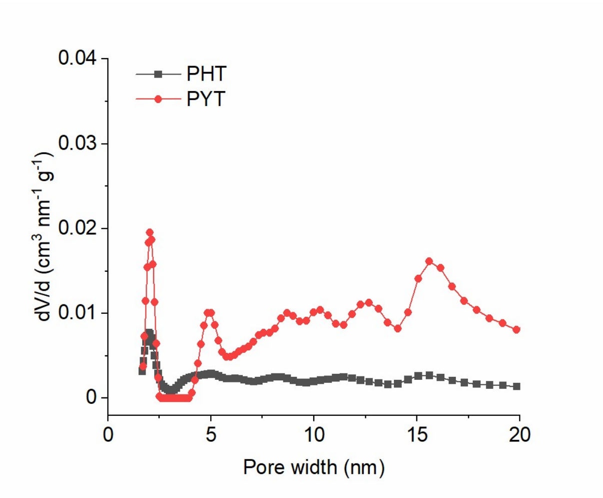

Supplementary Figure 5. Pore size distribution in CTFs.

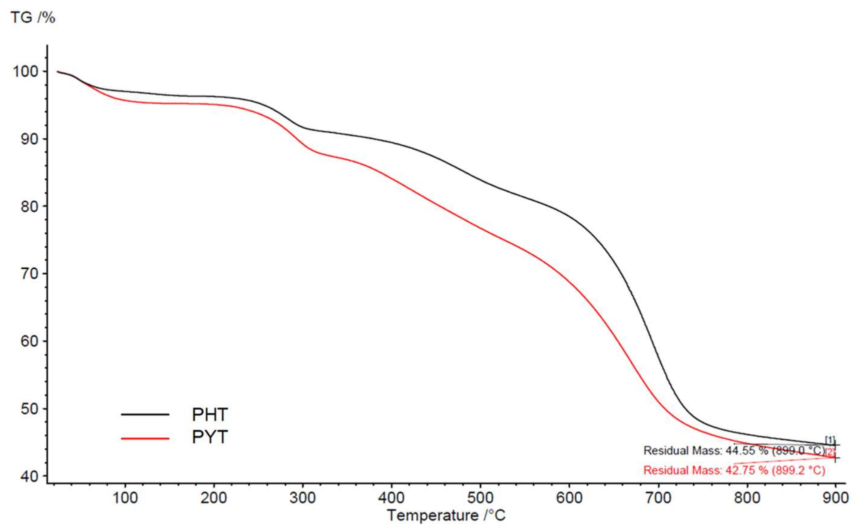

Supplementary Figure 6. TGA curves of CTFs.

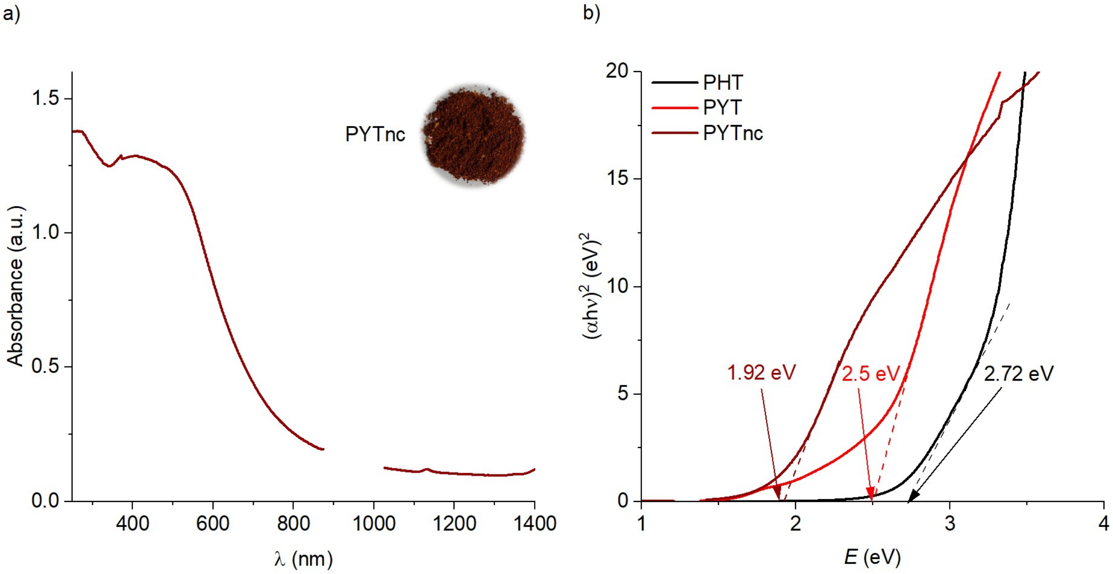

Supplementary Figure 7. Spectroscopic characterization of CTFs. a) DRUV-vis absorption spectrum of PYTnc. Some discontinuous data points caused by the change in detector at ~900 nm were removed. b) Tauc plots of PHT, PYT and PYTnc; π–π* transitions were taken as primary to determine optical band gap (E_g) in the materials.

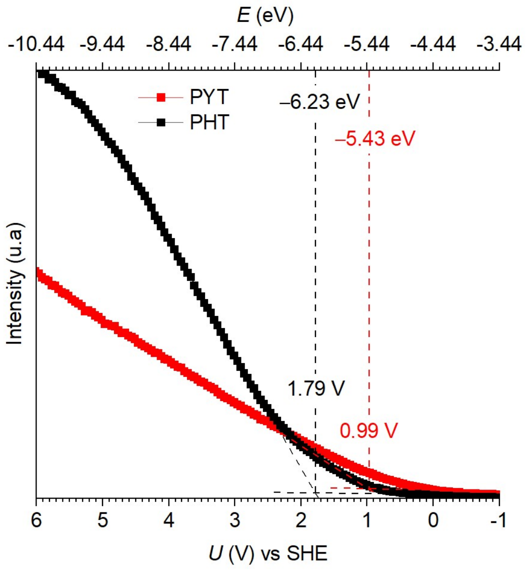

Supplementary Figure 8. Ultraviolet photoelectron spectra (UPS) of CTFs. Upper scale is in eV versus vacuum level (physical scale), bottom scale is in V versus SHE (electrochemical scale).

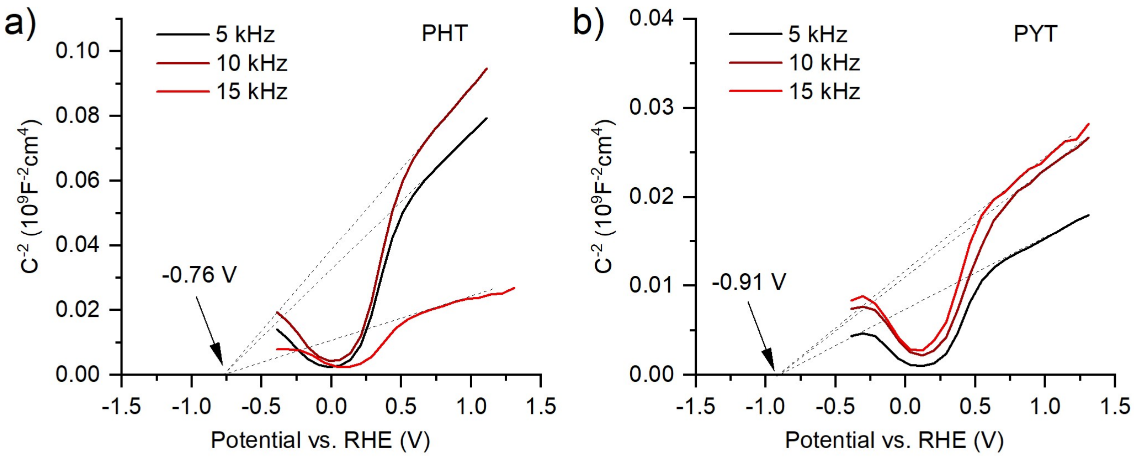

Supplementary Figure 9. Mott-Schottky plots. a) PHT. b) PYT.

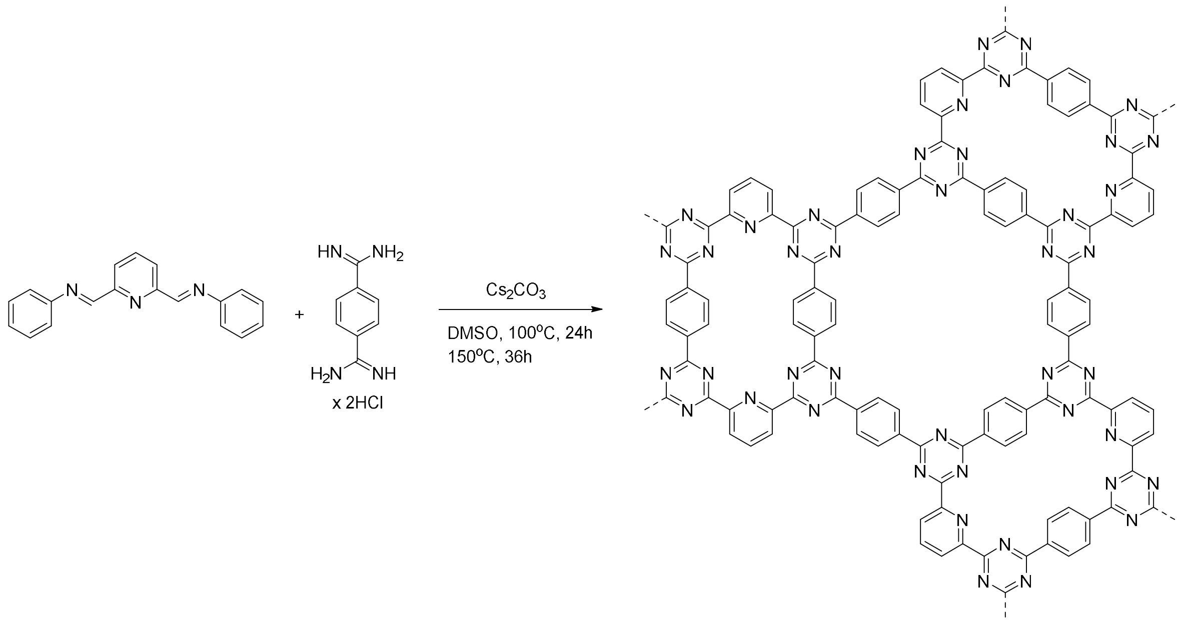

Supplementary Figure 10. Synthesis and ideal structure of PYTnc.

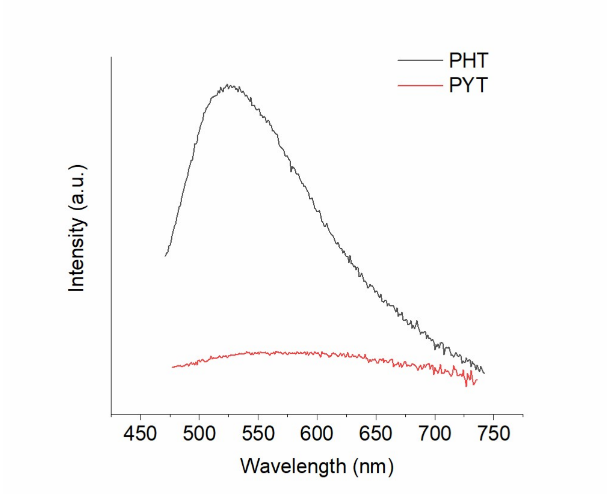

Supplementary Figure 11. PL spectra of PHT and PYT upon excitation at 450 nm.

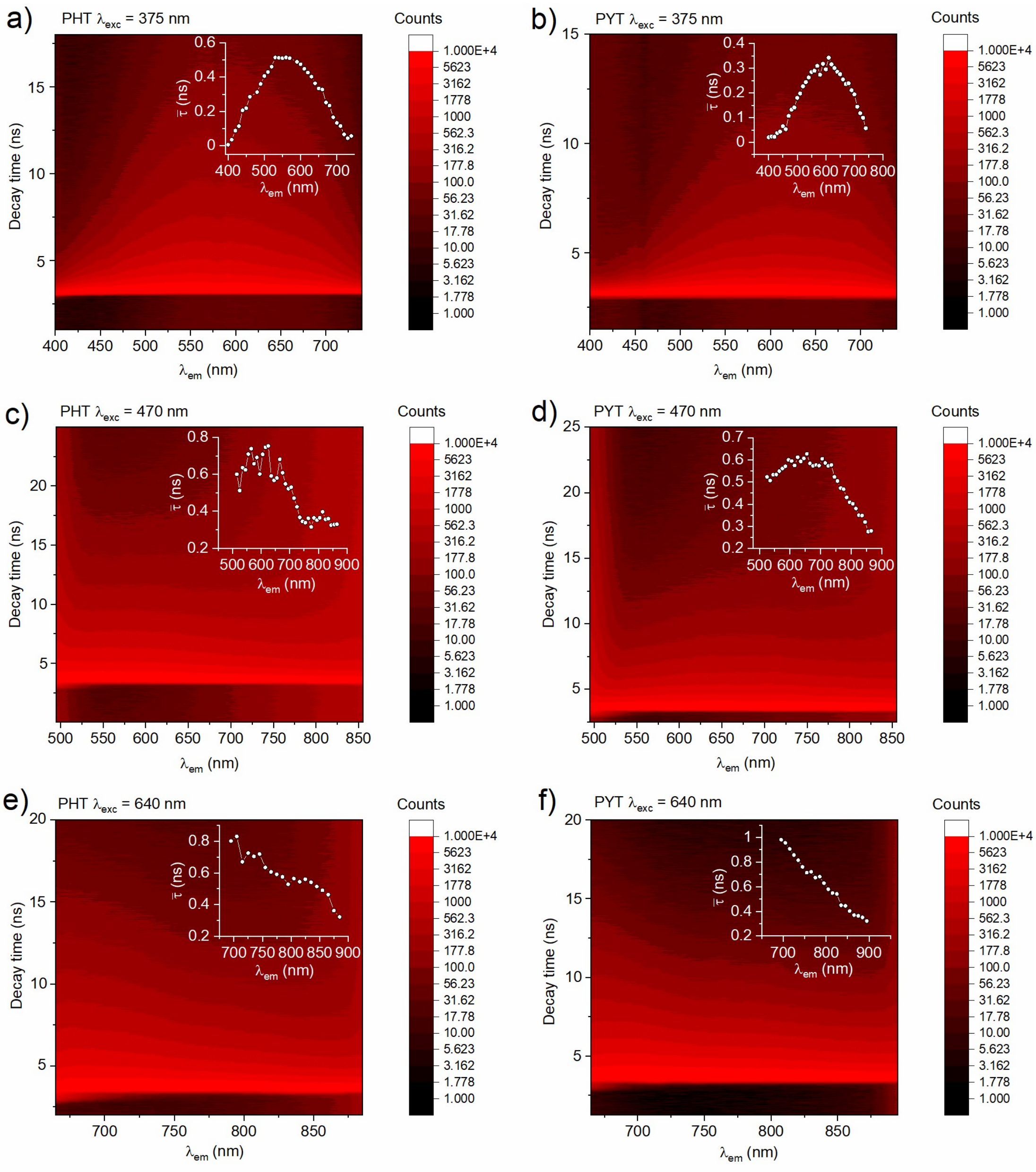

Supplementary Figure 12. 2D time-resolved (TR)-PL spectra of the CTFs upon excitation at λ_{exc} = 375, 470 and 640 nm with emission in the range λ_{em} = 400-900 nm. a) PHT PL decay upon excitation at λ_{exc} = 375 nm. b) PYT PL decay upon excitation at λ_{exc} = 375 nm. c) PHT PL decay upon excitation at λ_{exc} = 470 nm. d) PYT PL decay upon excitation at λ_{exc} = 470 nm. e) PHT PL decay upon excitation at λ_{exc} = 640 nm. f) PYT PL decay upon excitation at λ_{exc} = 640 nm. Insets show the corresponding $\bar{\tau}$ of the CTFs.

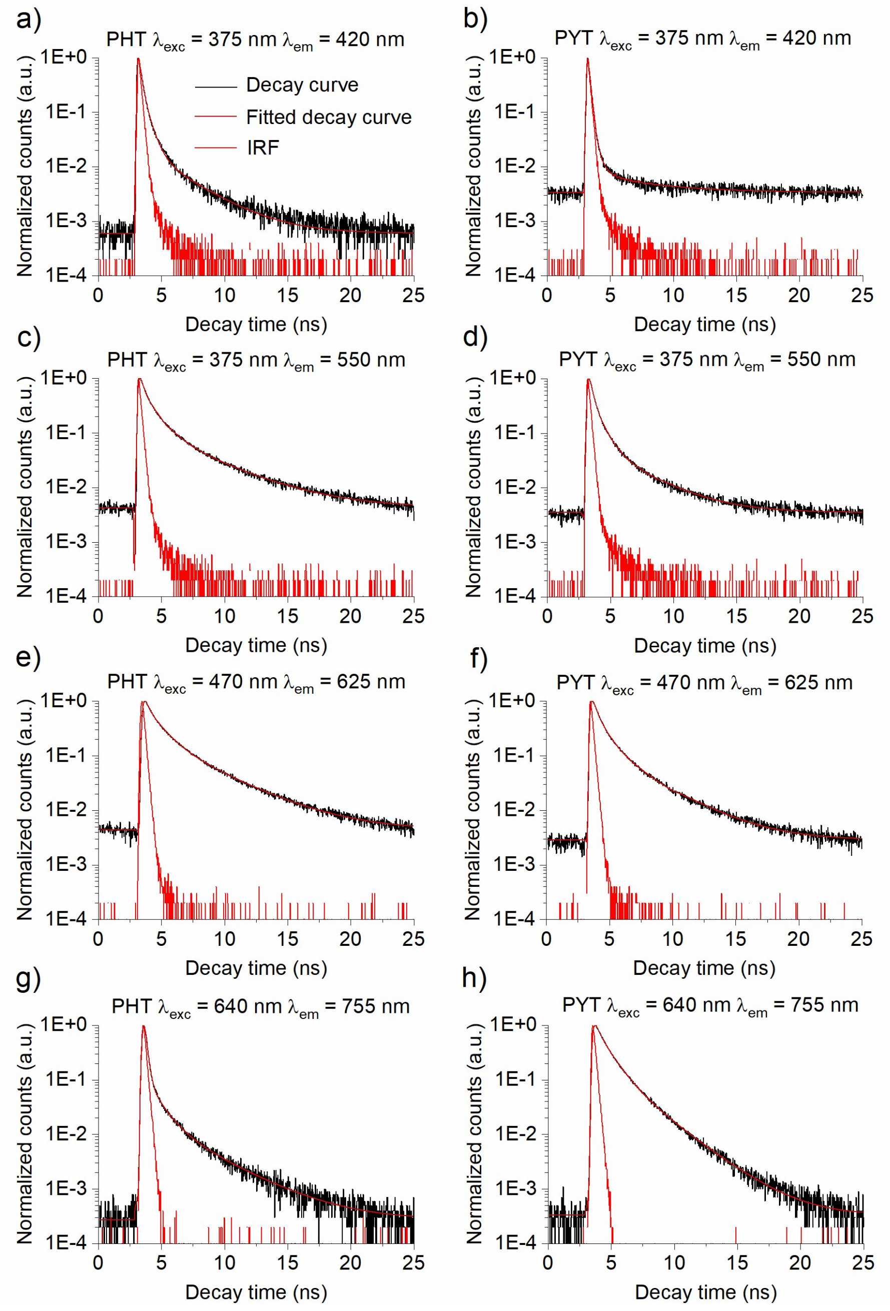

Supplementary Figure 13. Example of the PL decay curves with instrument response function (IRF). a) PHT PL decay monitored at λ_{em} = 420 nm upon excitation at λ_{exc} = 375 nm. b) PYT PL decay monitored at λ_{em} = 420 nm upon excitation at λ_{exc} = 375 nm. c) PHT PL decay monitored at λ_{em} = 550 nm upon excitation at λ_{exc} = 375 nm. d) PYT PL decay monitored at λ_{em} = 550 nm upon excitation at λ_{exc} = 375 nm. e) PHT PL decay monitored at λ_{em} = 625 nm upon excitation at λ_{exc} = 470 nm. f) PYT PL decay monitored at λ_{em} = 625 nm upon excitation at λ_{exc} = 470 nm. g) PHT PL decay monitored at λ_{em} = 755 nm upon excitation at λ_{exc} = 640 nm. h) PYT PL decay monitored at λ_{em} = 755 nm upon excitation at λ_{exc} = 640 nm.

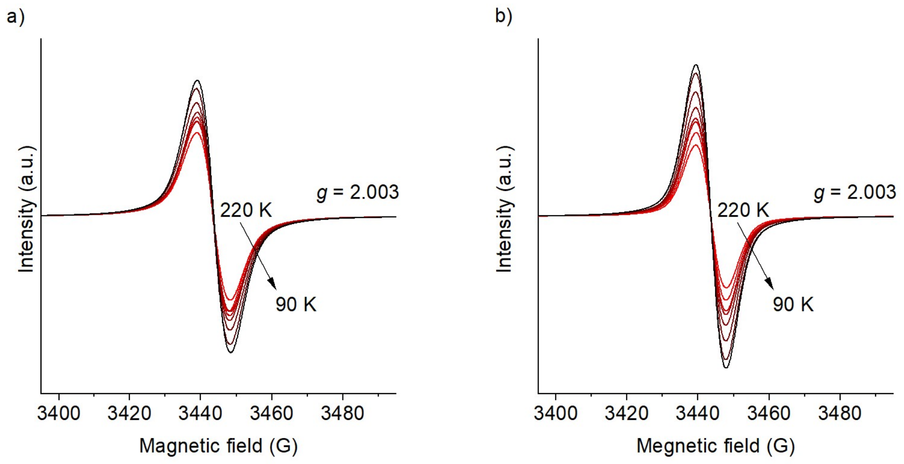

Supplementary Figure 14. Low-temperature EPR spectra of CTFs. a) PHT in dark. b) PYT in dark. Data were fitted. The g-factor of each EPR spectrum is given on the graph.

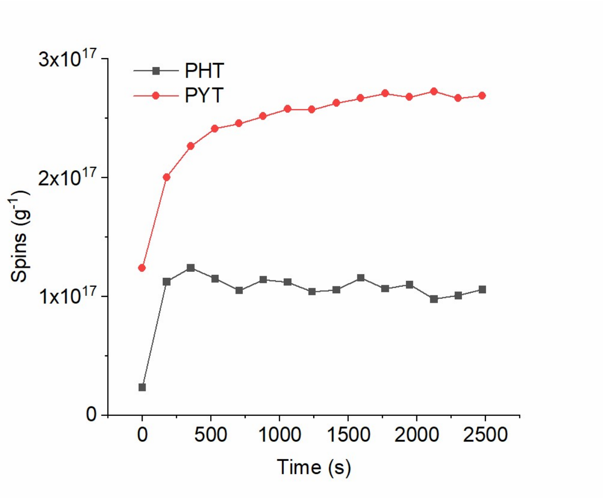

Supplementary Figure 15. Specific concentration of spins in PHT and PYT over irradiation time.

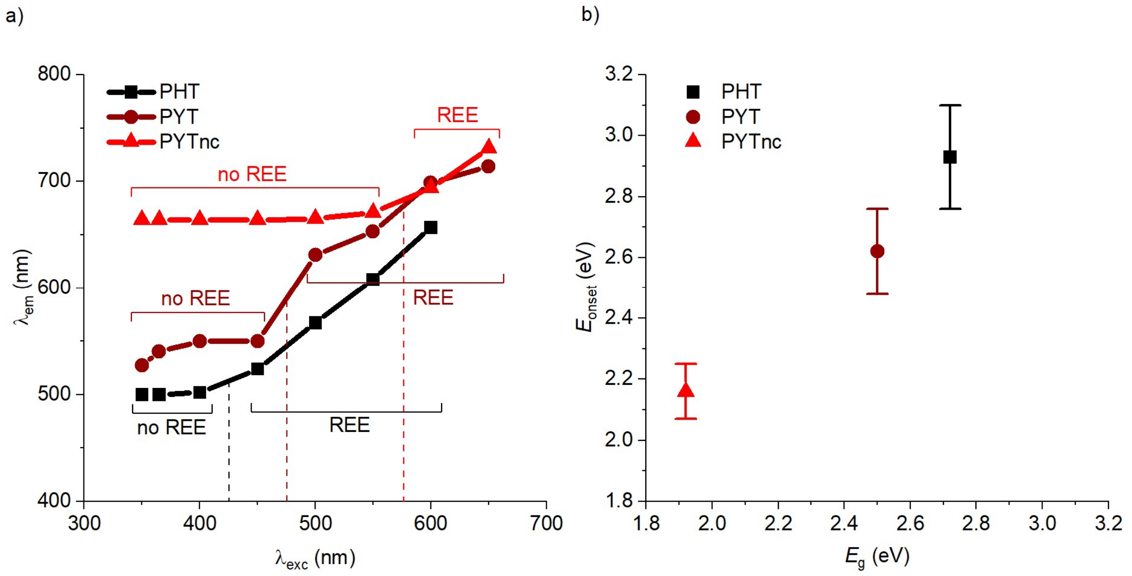

Supplementary Figure 16. Correlation between REE and E_g in CTFs. a) Plot of emission maximum (λ_{em}) versus excitation wavelength (λ_{exc}). Note two regions for each curve: no REE (position of the emission maximum peak remains nearly constant regardless the λ_{exc}) and REE (position of the emission maximum peak follows the λ_{exc}). b) Correlation between REE onset and optical gap defined by π–π* transitions. REE onset was determined as average of two adjacent λ_{exc} values in panel (a) at which REE and no REE was observed followed by conversion into eV using Planck-Einstein relation. Thus, error bars correspond to std. dev. (n = 2).

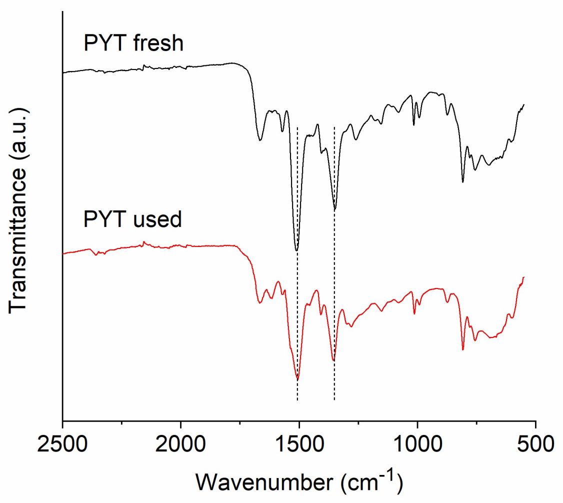

Supplementary Figure 17. FT-IR spectra of fresh PYT and PYT recovered after photocatalytic bromination of anisole.

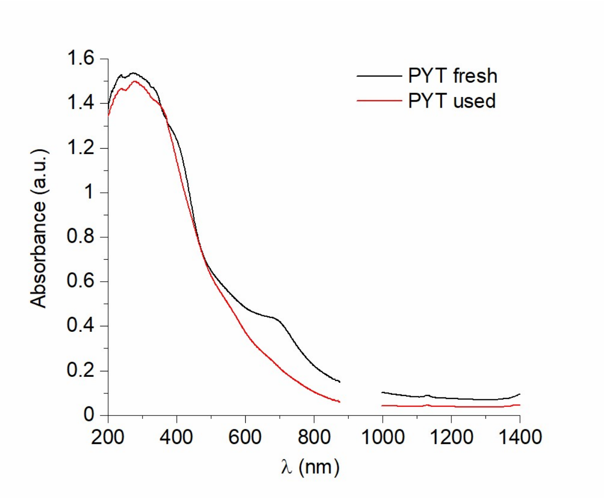

Supplementary Figure 18. Optical absorbance spectra of fresh PYT and PYT recovered after photocatalytic bromination of anisole. Some discontinuous data points caused by the change in detector at ~900 nm were removed. Partial bleaching of n→π* intraband states is ascribed to protonation of nitrogen lone pairs under highly acidic conditions in photocatalytic bromination of anisole.

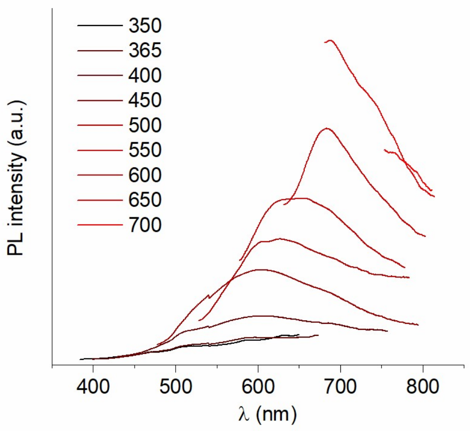

Supplementary Figure 19. PL spectra of PYT recovered after the photocatalytic bromination of anisole upon a range of λ_{exc}.

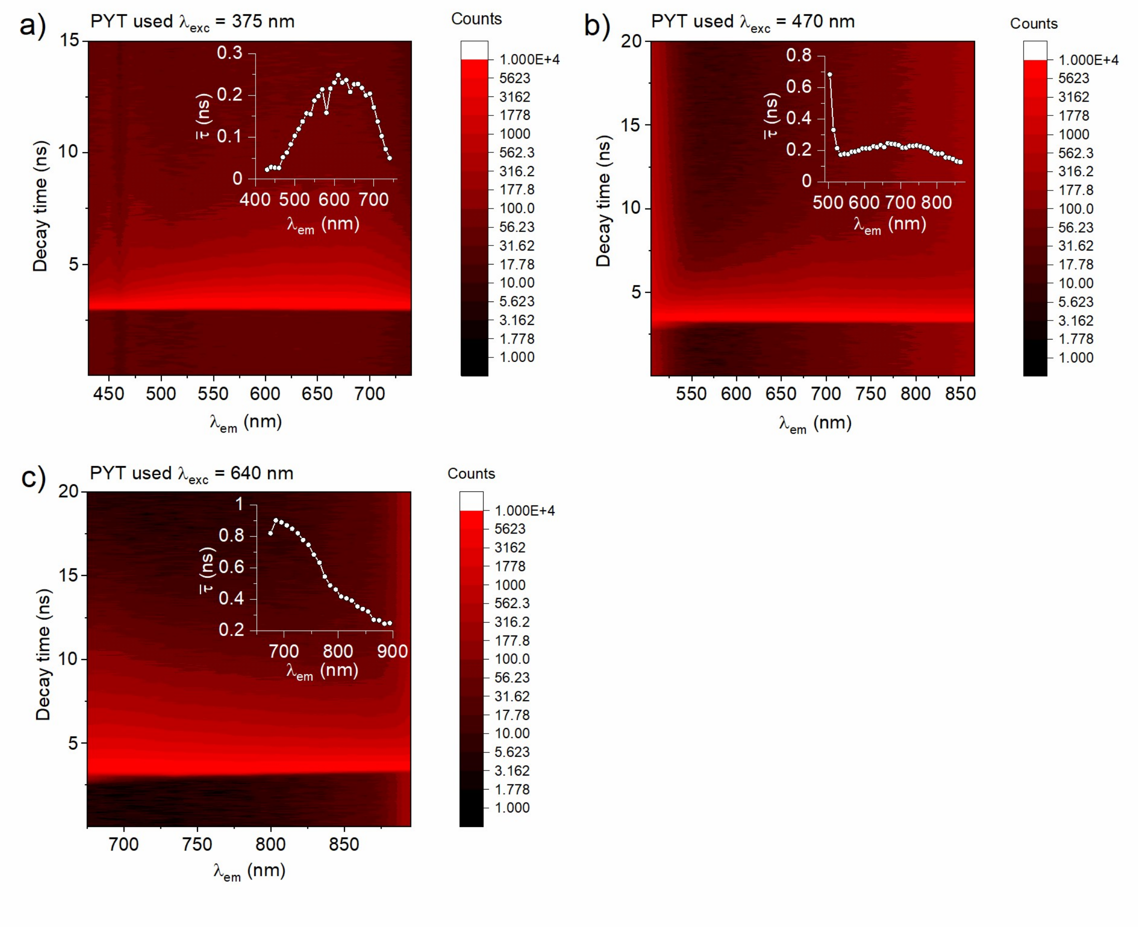

Supplementary Figure 20. 2D time-resolved (TR)-PL spectra of PYT recovered after photocatalytic bromination of anisole excited at λ_{exc} = 375, 470 and 640 nm with emission at λ_{em} = 400-900 nm. Inset shows the corresponding $\bar{\tau}$ of the CTFs. a) PYT PL decay upon excitation at λ_{exc} = 375 nm. b) PYT PL decay upon excitation at λ_{exc} = 470 nm. c) PYT PL decay upon excitation at λ_{exc} = 640 nm.

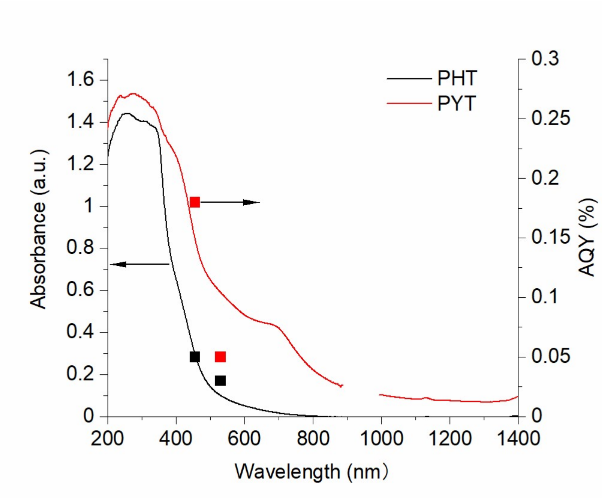

Supplementary Figure 21. AQY of photocatalytic bromination of anisole with the CTFs and the corresponding optical absorbance spectra.

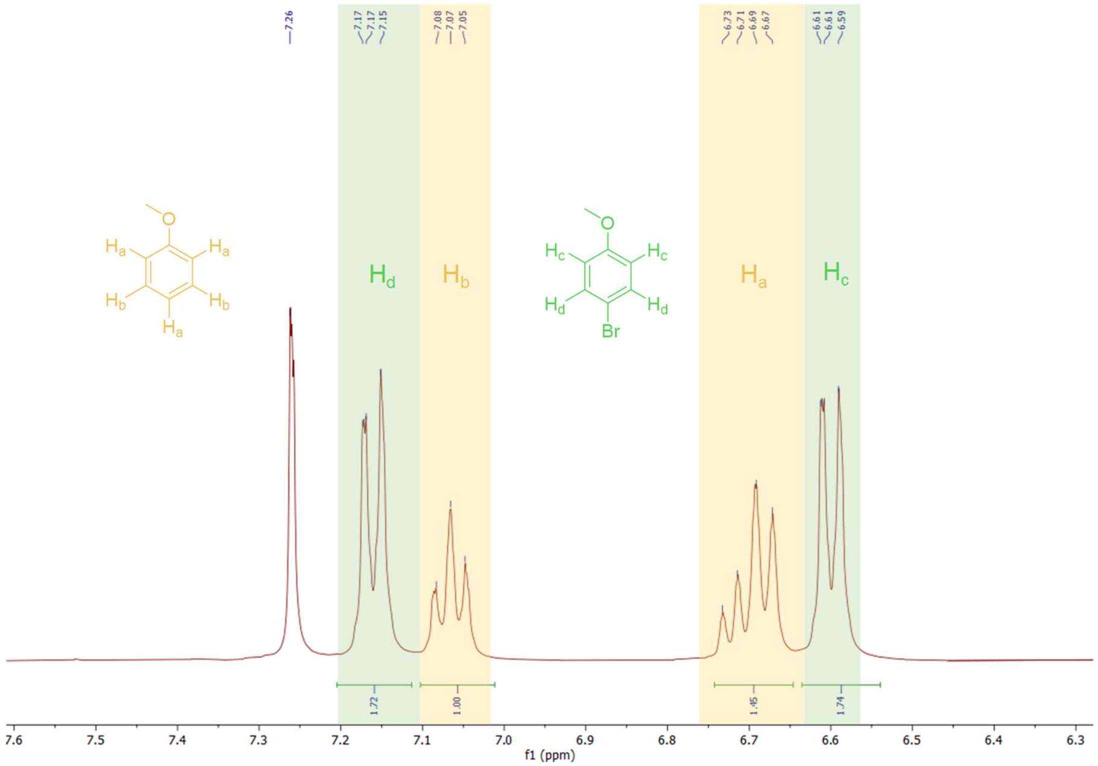

Supplementary Figure 22. An exemplary ^{1}H NMR spectrum of anisole bromination reaction mixture. Signals of hydrogen atoms in anisole are highlighted with yellow color, while those in 4-bromoanisole are highlighted with green color.

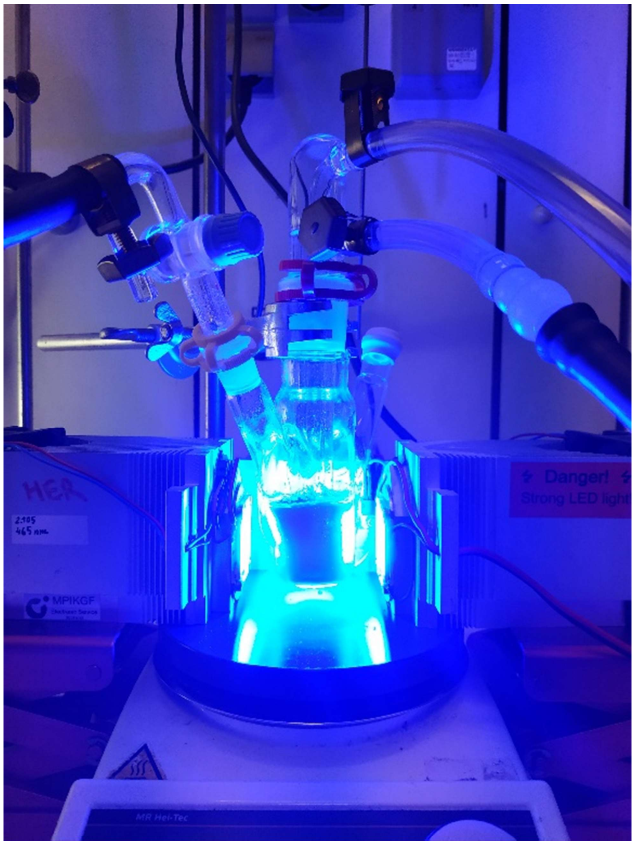

Supplementary Figure 23. An image of the setup used for oxidative bromination performed on 6 mmol scale of anisole.

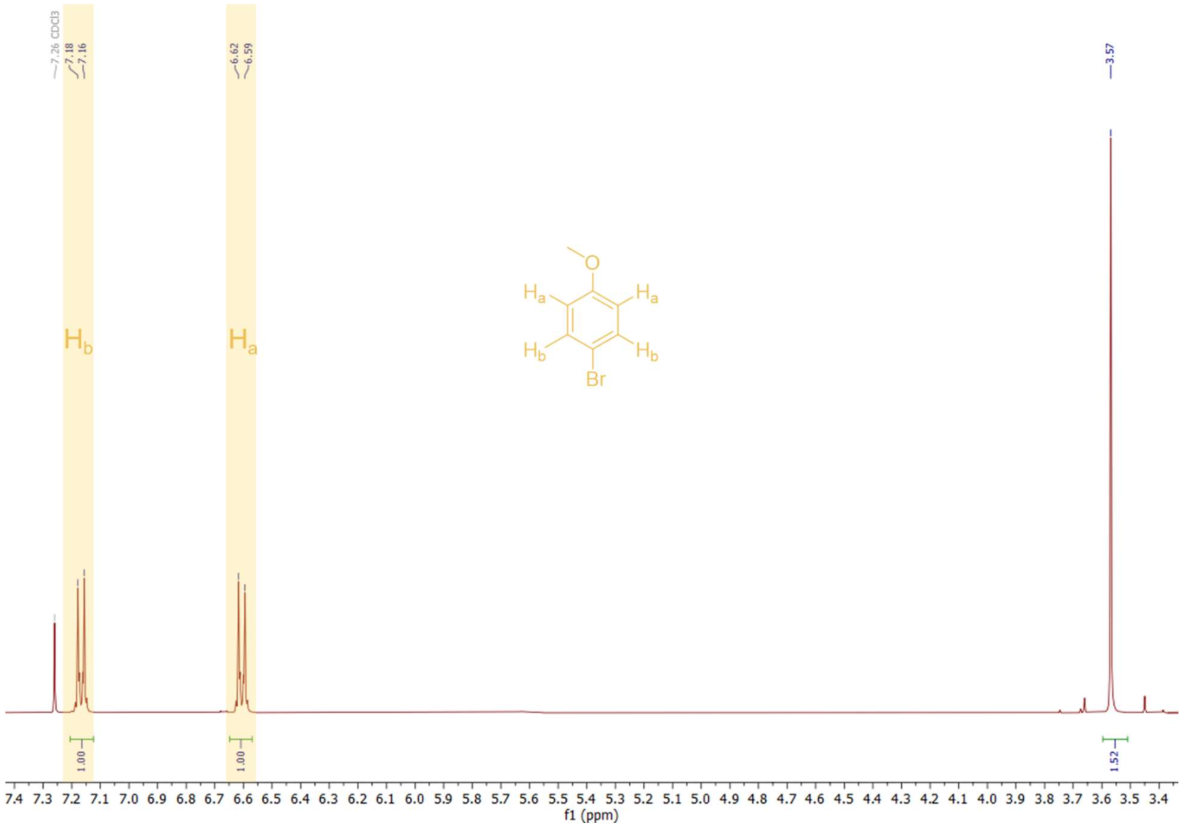

Supplementary Figure 24. ^{1}H NMR spectrum of the reaction mixture with anisole as substrate.

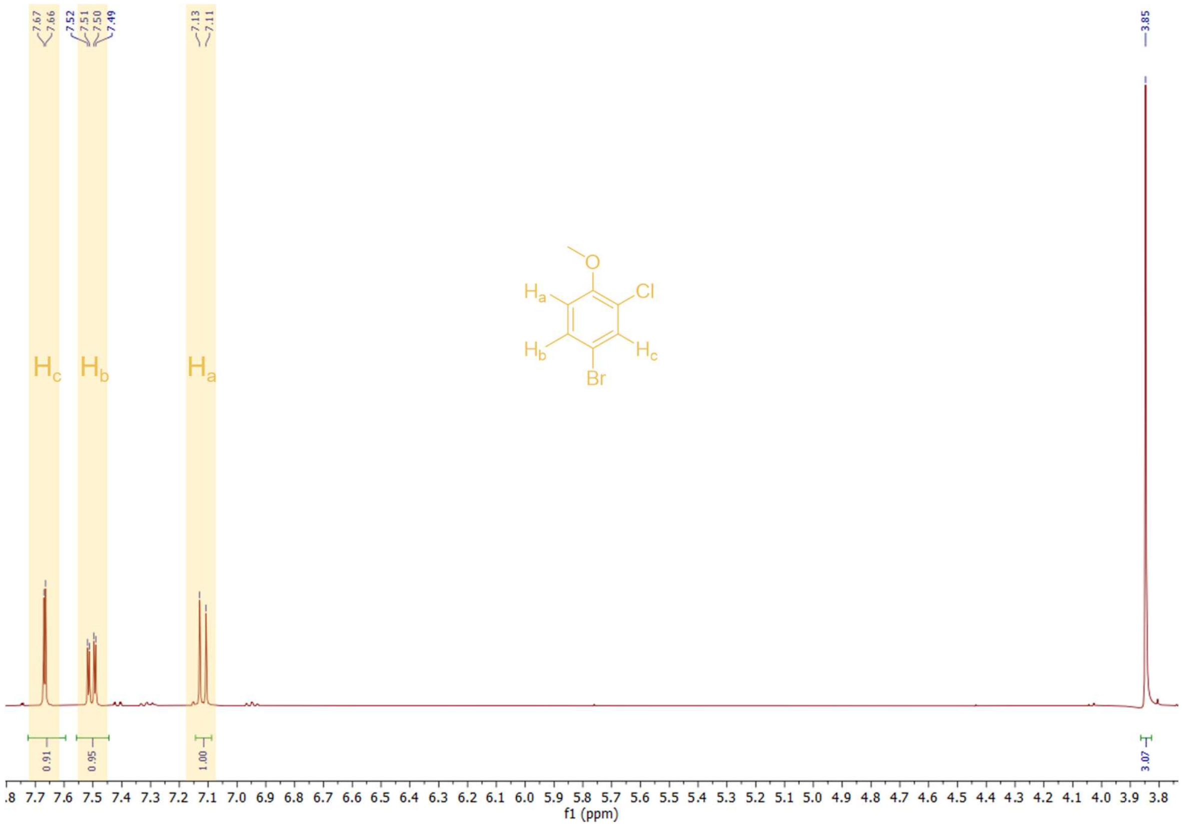

Supplementary Figure 25. ^{1}H NMR spectrum of the reaction mixture with 4-bromo-2-chloroanisole as substrate.

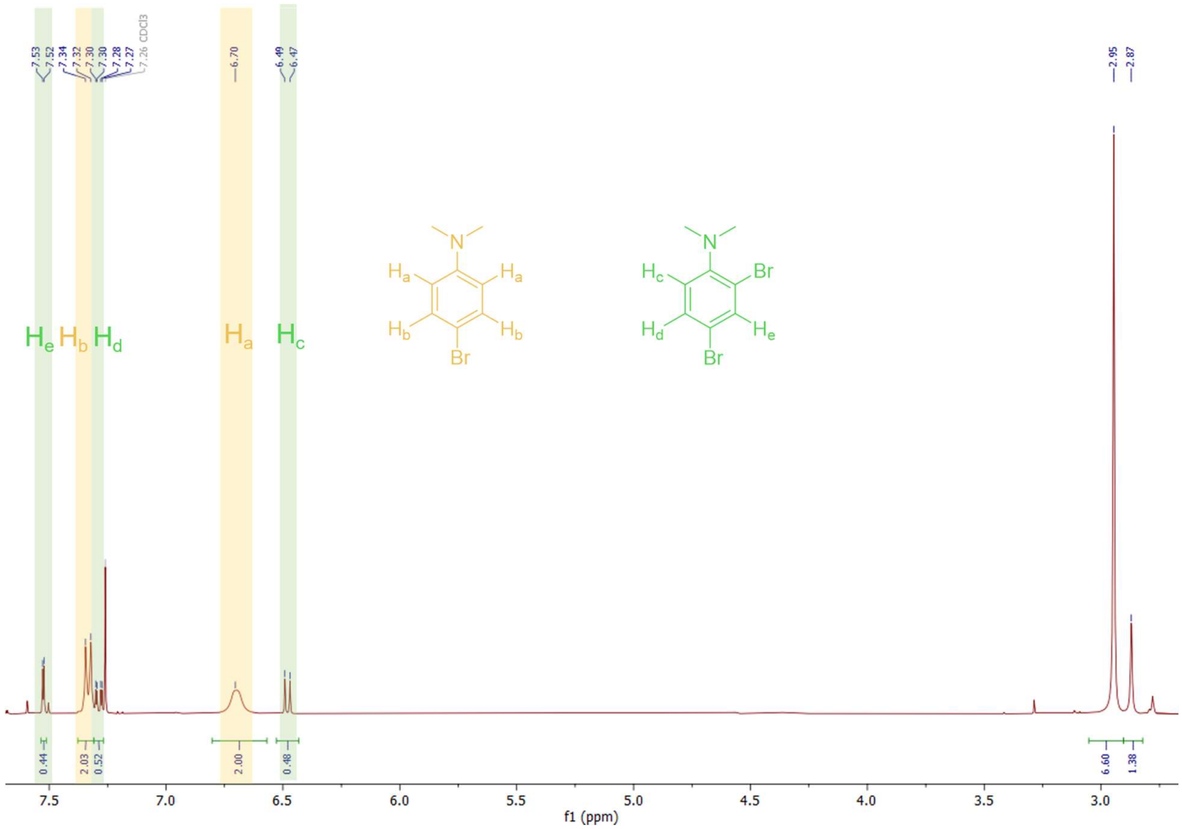

Supplementary Figure 26. ^{1}H NMR spectrum of the reaction mixture with N,N-dimethylaniline as substrate. Signals of hydrogen atoms in 4-bromo-N,N-dimethylaniline are highlighted with yellow color, while those in 2,4-dibromo-N,N-dimethylaniline are highlighted with green color.

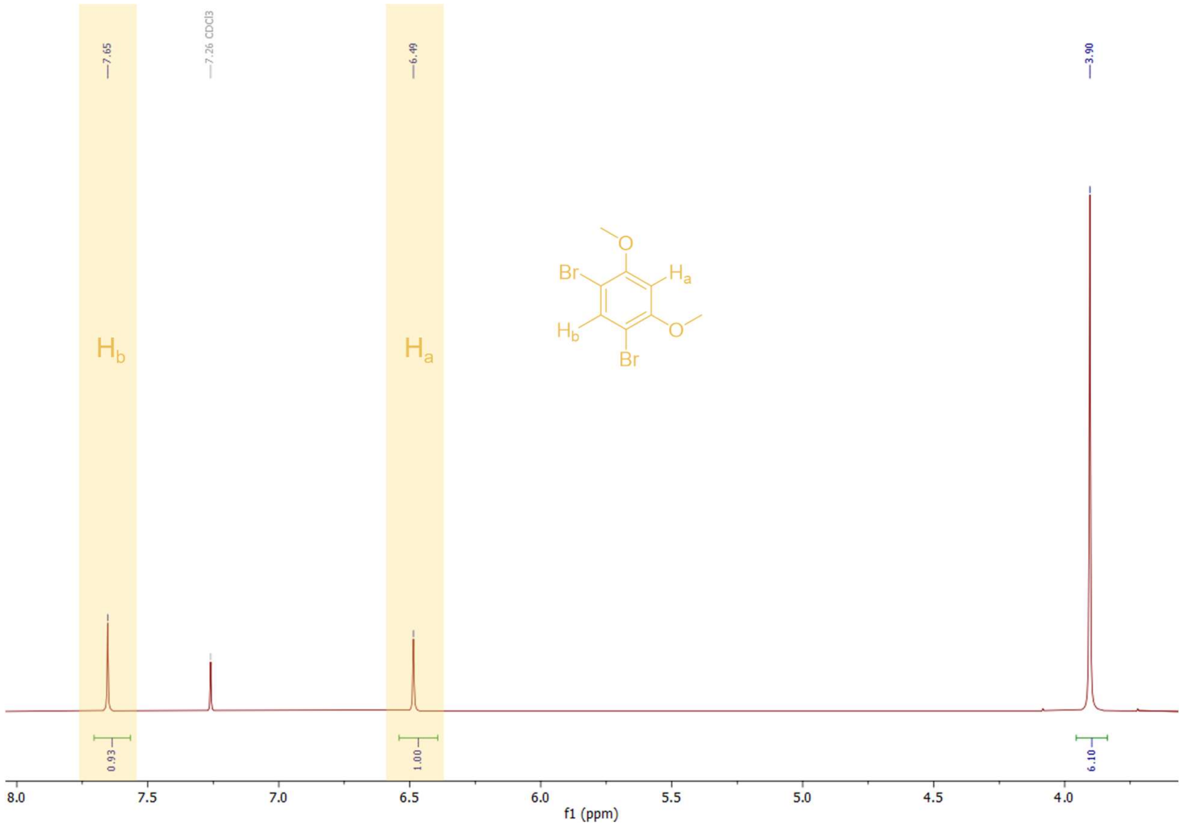

Supplementary Figure 27. ^{1}H NMR spectrum of the reaction mixture with 1,3-dimethoxybenzene as substrate.

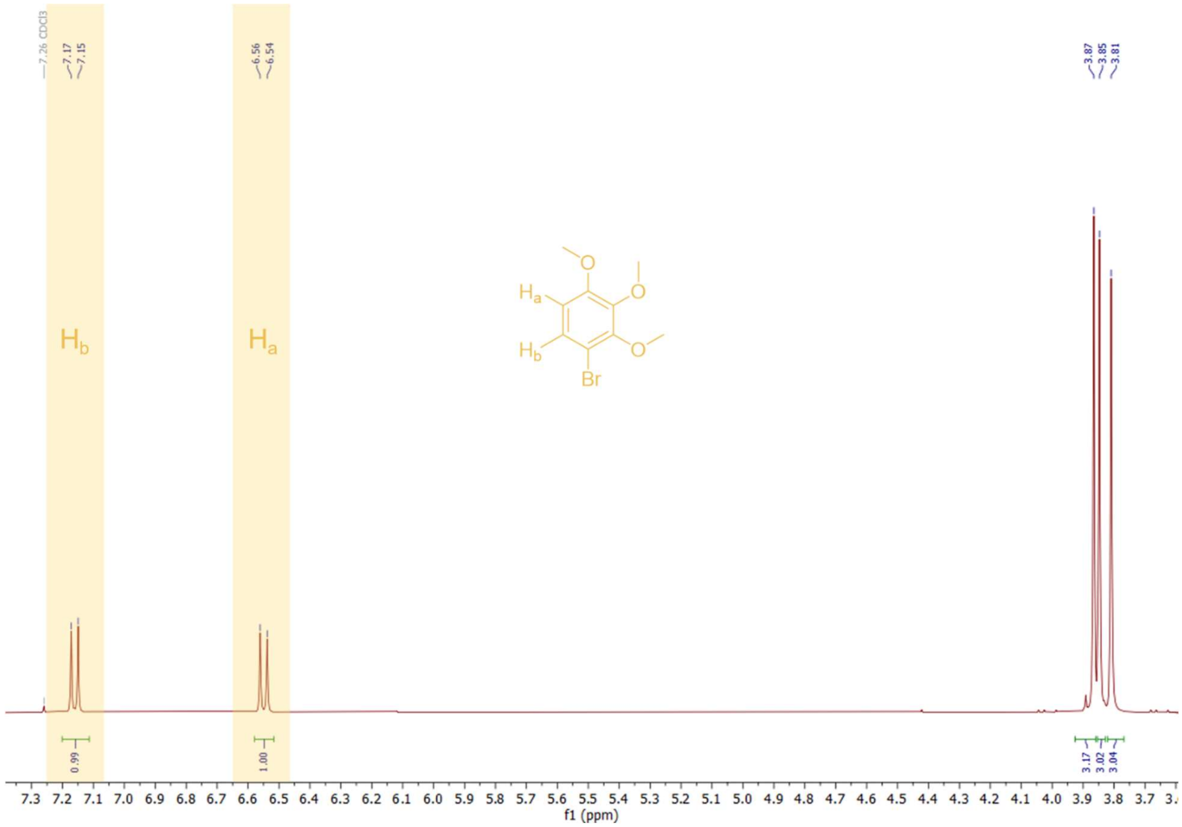

Supplementary Figure 28. ^{1}H NMR spectrum of the reaction mixture with 1,2,3-trimethoxybenzene as substrate.

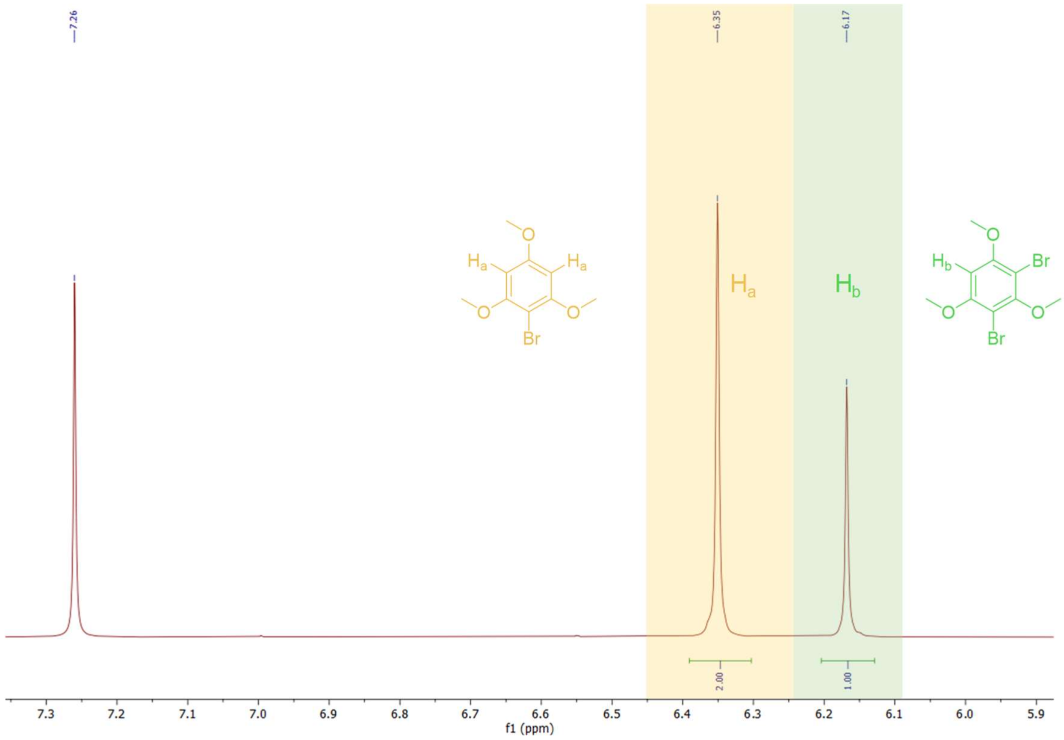

Supplementary Figure 29. ^{1}H NMR spectrum of the reaction mixture with 1,3,5-trimethoxybenzene as substrate. Signals of hydrogen atoms in 4-bromo-1,3,5-trimethoxybenzene are highlighted with yellow color, while those in 2,4-dibromo-1,3,5-trimethoxybenzene are highlighted with green color.

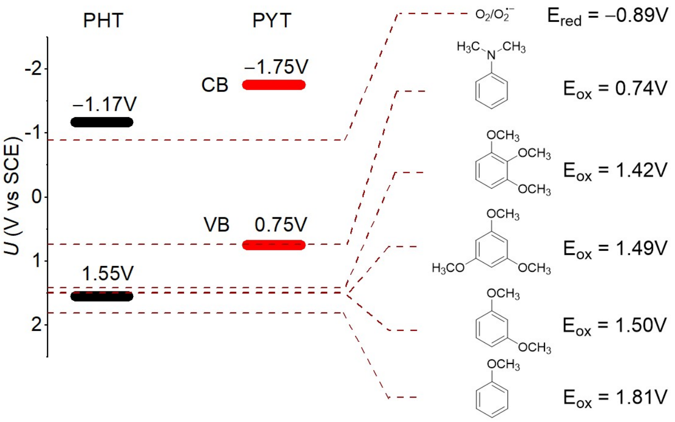

Supplementary Figure 30. Potentials of VB and CB in PHT and PYT and redox potentials of organic molecules. Oxidation potentials of aniline,[4] 1,2,3-trimethoxybenzene,[10] 1,3,5-trimethoxybenzene,[10] 1,3-dimethoxybenzene[4] and anisole[4] and reduction potential of O_2 to $O_2^{-\bullet}$ (in acetonitrile)[11] in electrochemical scale (vs SCE). Potentials of VB and CB in PHT and PYT were calculated by converting CB and VB energy levels (eV, shown in Figure 3b) using equation:

$$U = -k\cdot(E + U_{AVS/SHE}) - U_{SCE/SHE} \quad (9)$$

where E – CB or VB energy level, eV; k – conversion factor, 1 V·eV^{-1}; $U_{AVS/SHE}$ – “potential” of standard hydrogen electrode expressed in physical scale, +4.44 eV; $U_{SCE/SHE}$ – potential of SCE versus SHE, +0.244 V.[12]

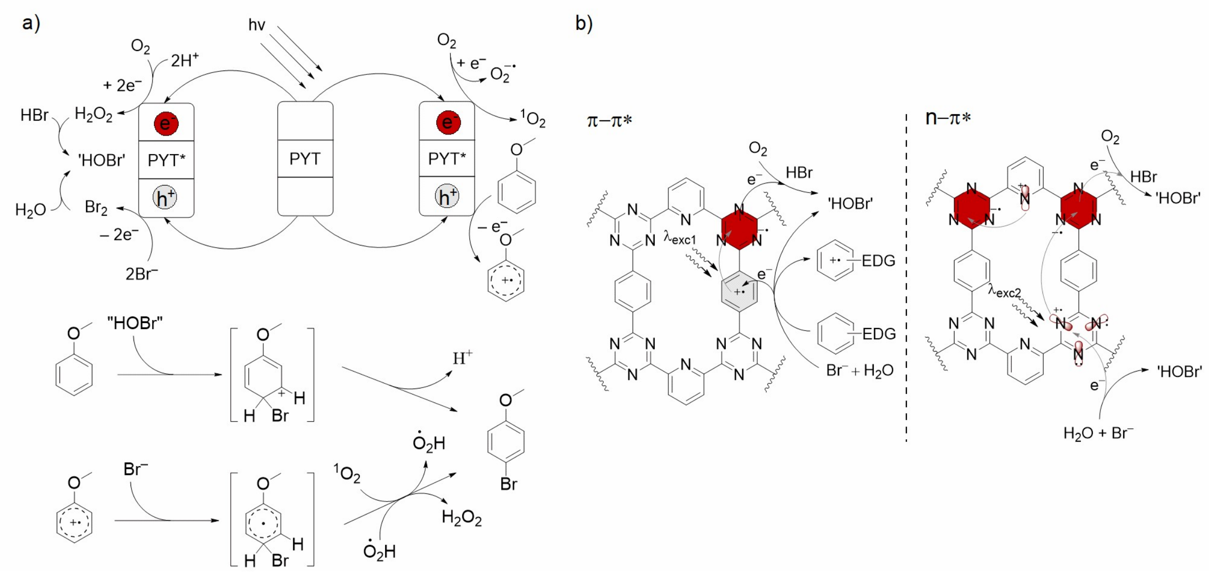

Supplementary Figure 31. A summary of a mechanism of photocatalytic oxidative bromination that involves π–π* and n–π* excited states of CTFs. a) Proposed reaction mechanism of the photocatalytic bromination of anisole using PYT as the photocatalyst. b) Chromoselective generation of more oxidative (upon π→π* transitions) and less oxidative (upon n→π* transitions) sites in PYT. VB is assumed to be composed of p orbitals of p-phenylene as electron richer part of the CTF, while CB – p orbitals of triazine as electron deficient part of the CTF.

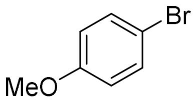

3e

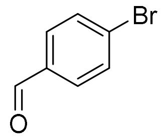

3f

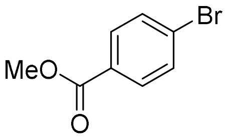

3g

Supplementary Figure 32. A list of arylbromides that did not give the C–N coupling products. The starting materials have been recovered. Condition were adapted from the reference [5]. Arylhalide (0.05 mmol), PHT (12 mg), pyrrolidine (7.4 µL, 0.09 mmol), $NiBr_2$·glyme (0.8 mg, 0.0025 mmol), DABCO (12.3 mg, 0.11 mmol), N,N-dimethylacetamide (1 mL), Light 302 mW cm^{-2}, 168 h. Yield and conversion (in parentheses) determined via GC-MS.

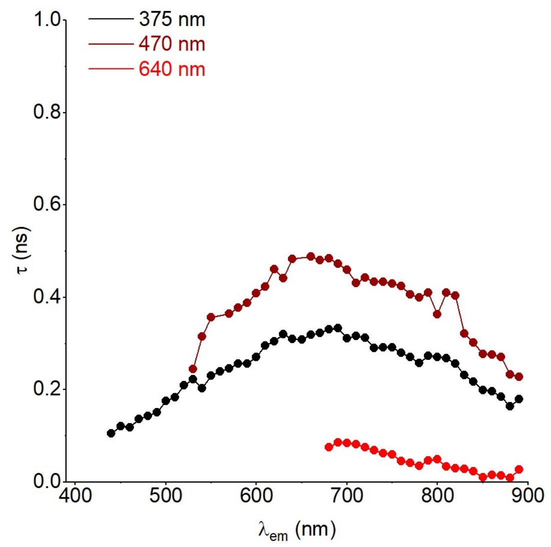

Supplementary Figure 33. Amplitude average lifetime of PYTnc obtained with λ_{exc} = 375, 470 and 640 nm and λ_{em} = 400-900 nm.

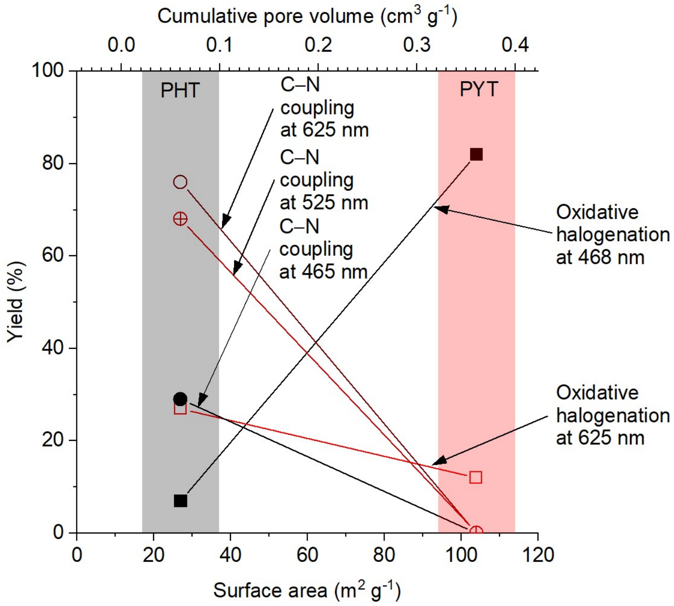

Supplementary Figure 34. Correlation between the surface area and cumulative pore volume of the CTFs, yield of 4-bromoanisole **2a** and the product of C–N cross-coupling **5a**. Grey bars label data points obtained for PHT, pink bars – PYT.

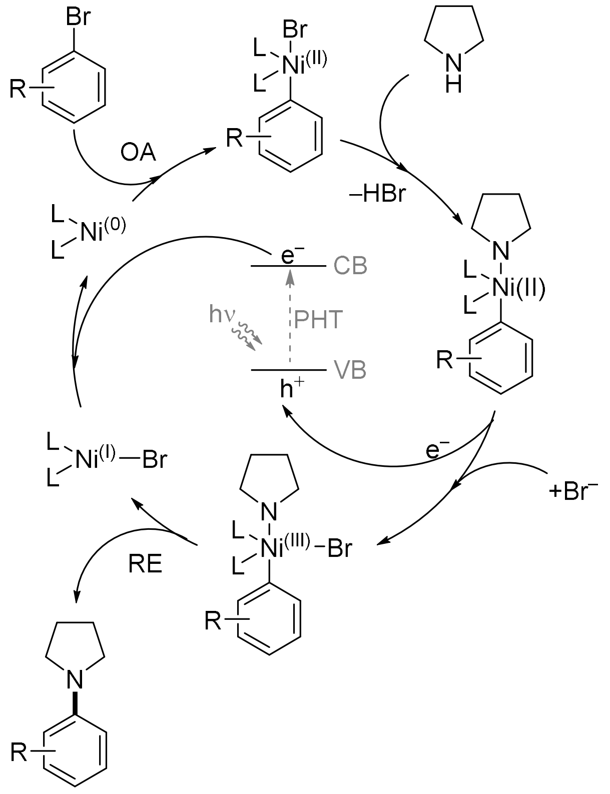

Supplementary Figure 35. A tentative mechanism of Ni-dual photocatalytic C–N cross coupling mediated by PHT. Adapted from reference [6]. RE – denotes "reductive elimination", OA – "oxidative addition".

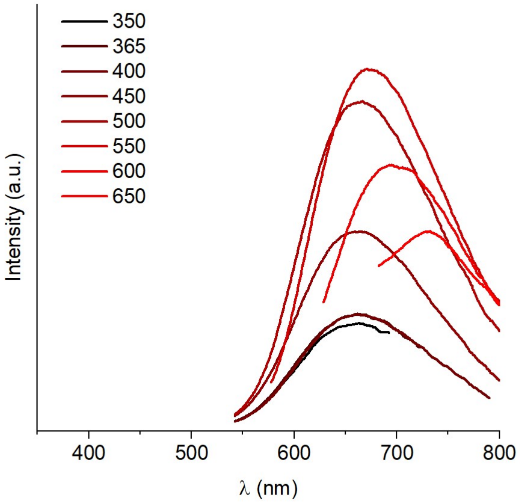

Supplementary Figure 36. PL spectra of PYTnc acquired at λ_{exc} 350, 365, 400, 450, 500, 550, 600 and 650 nm.

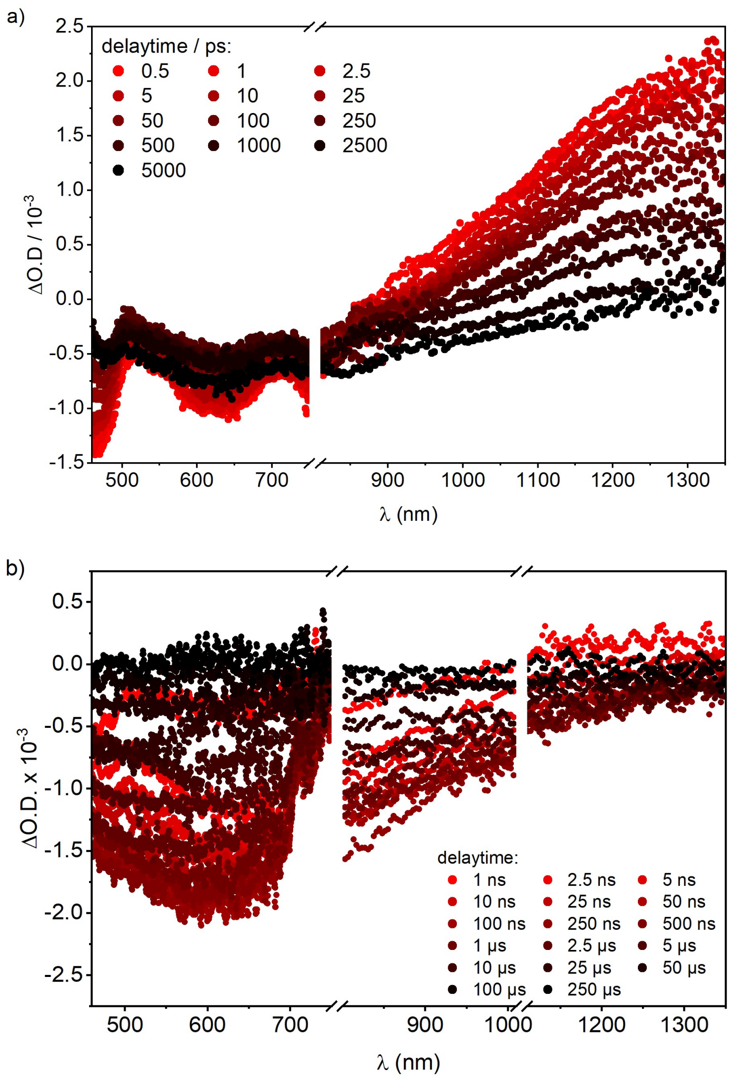

Supplementary Figure 37. Transient absorption spectroscopy of PYT. Differential absorption changes in the visible and near-infrared (nIR) region of the electromagnetic spectrum obtained upon sub-picosecond (a) and nanosecond (b) pump-probe transient absorption experiments (387 nm / 2 µJ) of argon-purged dispersion of PYT in MeCN. Areas around 775 and 1064 nm are removed due to major contributions from the fundamental wavelength of the respective laser source used for white light generation or detector changes.

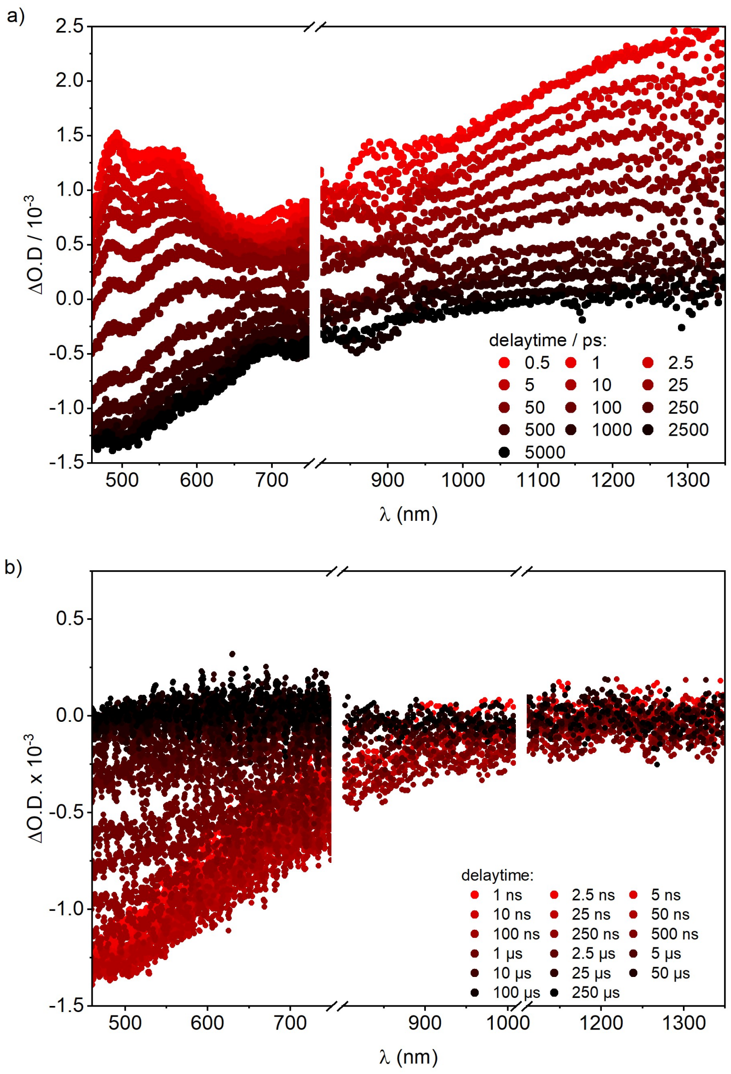

Supplementary Figure 38. Transient absorption spectroscopy of PYTnc. Differential absorption changes in the visible and near-infrared (nIR) region of the electromagnetic spectrum obtained upon sub-picosecond (a) and nanosecond (b) pump-probe transient absorption experiments (387 nm / 2 μJ) of argon-purged dispersion of PYTnc in MeCN. Areas around 775 and 1064 nm are removed due to major contributions from the fundamental wavelength of the respective laser source used for white light generation or detector changes.

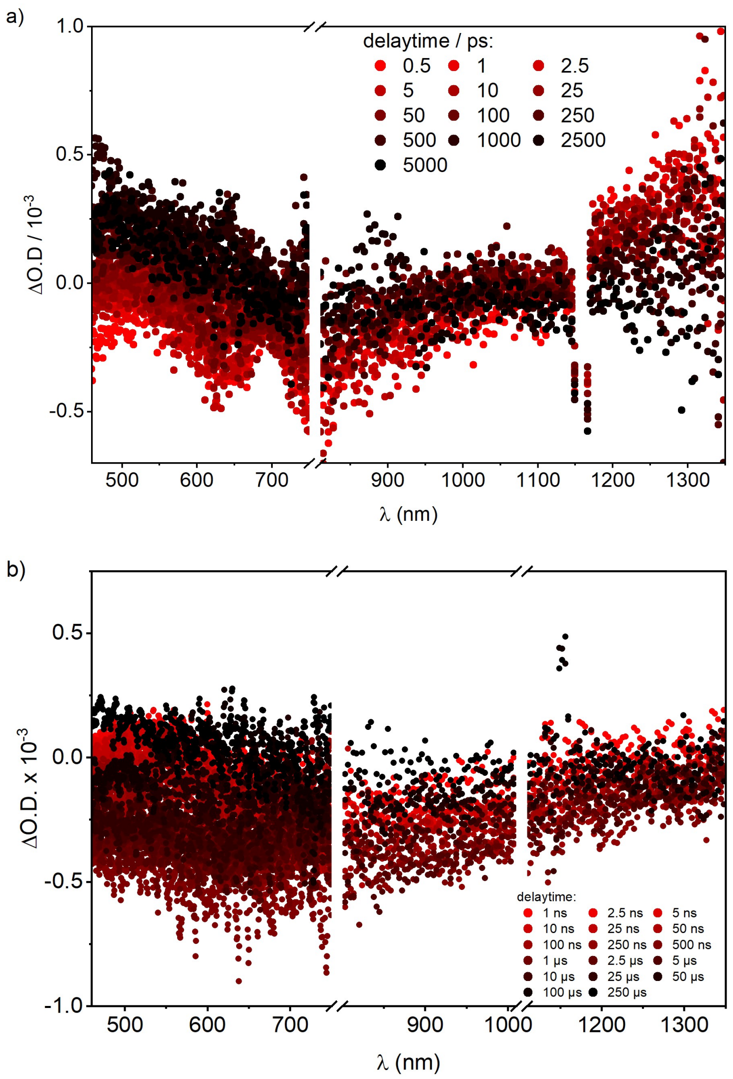

Supplementary Figure 39. Transient absorption spectroscopy of PHT. Differential absorption changes in the visible and near-infrared (nIR) region of the electromagnetic spectrum obtained upon sub-picosecond (a) and nanosecond (b) pump-probe transient absorption experiments (387 nm / 2 µJ) of argon-purged dispersion of PHT in MeCN. Areas around 775 and 1064 nm are removed due to major contributions from the fundamental wavelength of the respective laser source used for white light generation or detector changes.

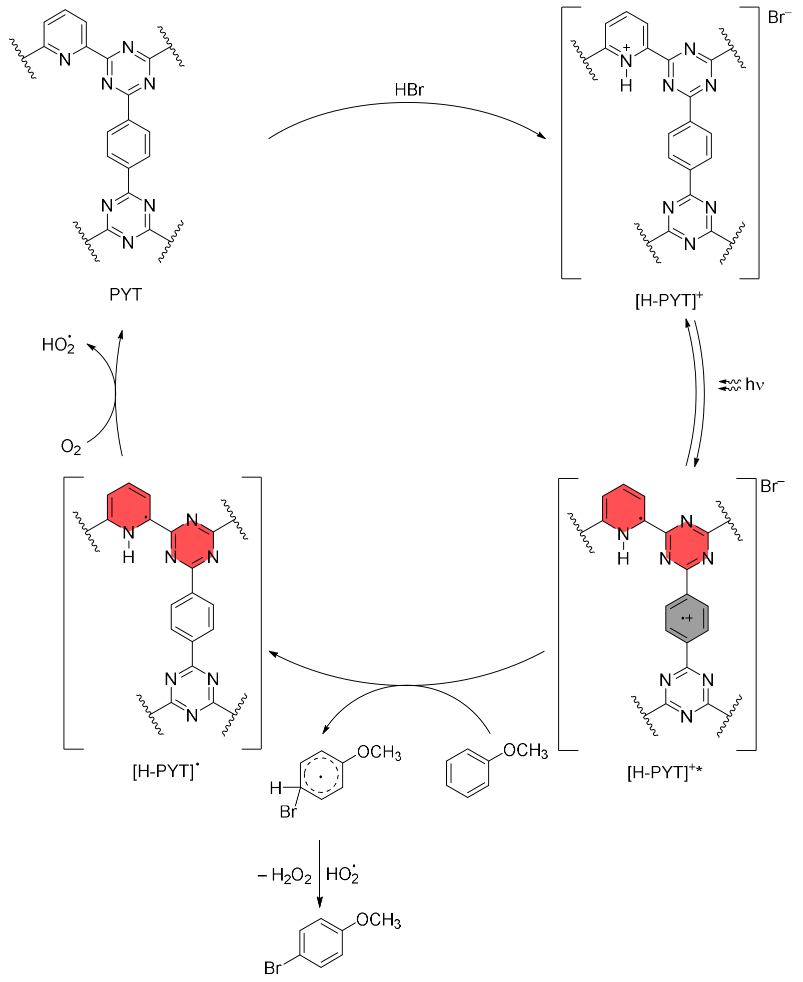

Supplementary Figure 40. Schematic mechanism of anisole oxidative bromination with PYT. In this figure only electron transfer is considered. Tentative local structure of PYT intermediates is shown. Upon excitation with light, electron is transferred from electron enriched part (p-phenylene units) to electron-deficient part (pyridine-2,6-diyl and/or triazine-linkers) of PYT.

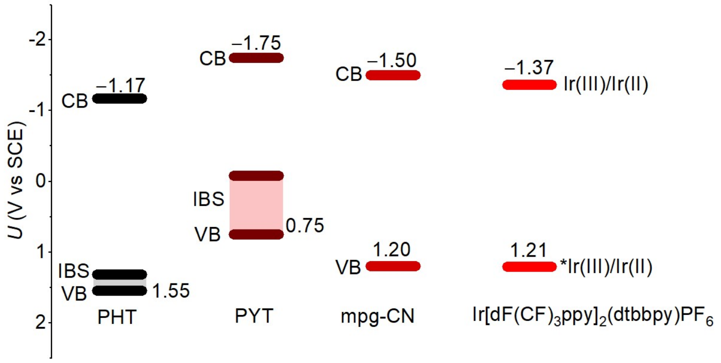

Supplementary Figure 41. Positions of band edges in semiconductors and redox potentials of molecular sensitizers. Positions of band edges in PHT, PYT (this work) and mpg-CN(reference [5]) and redox potentials of *Ir(III)/Ir(II) and I(III)/Ir(II) couples (reference [13],[6]). Intraband states (IBS) in PHT and PYT are schematically depicted based on the data shown in Figure 3.

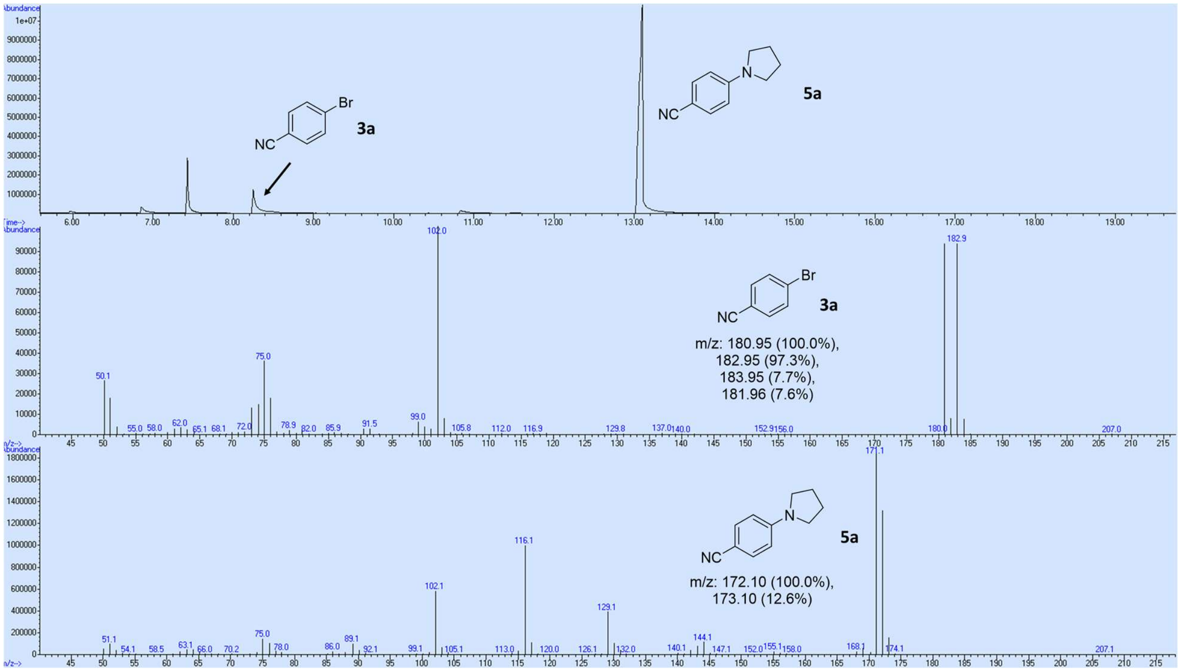

Supplementary Figure 42. Gas chromatogram of the reaction mixture and mass spectra of components using **3a** as the reagent. 4-bromobenzonitrile (0.05 mmol), PHT (12 mg), pyrrolidine (7.4 µL, 0.09 mmol), $NiBr_2$·glyme (0.8 mg, 0.0025 mmol), DABCO (12.3 mg, 0.11 mmol), N,N-dimethylacetamide (1 mL), Light 302 mW cm^{-2}, 168 h.

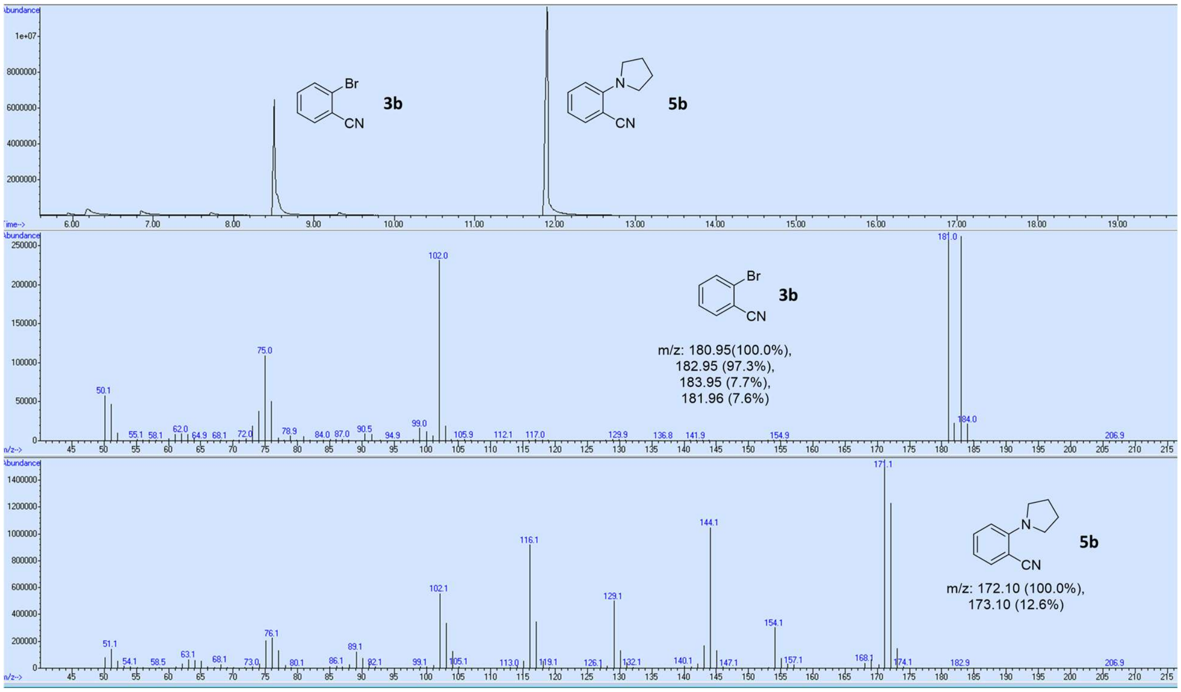

Supplementary Figure 43. Gas chromatogram of the reaction mixture and mass spectra of components using **3b** as the reagent. 2-bromobenzonitrile (0.05 mmol), PHT (12 mg), pyrrolidine (7.4 µL, 0.09 mmol), $NiBr_2$·glyme (0.8 mg, 0.0025 mmol), DABCO (12.3 mg, 0.11 mmol), N,N-dimethylacetamide (1 mL), Light 302 mW cm^{-2}, 168 h.

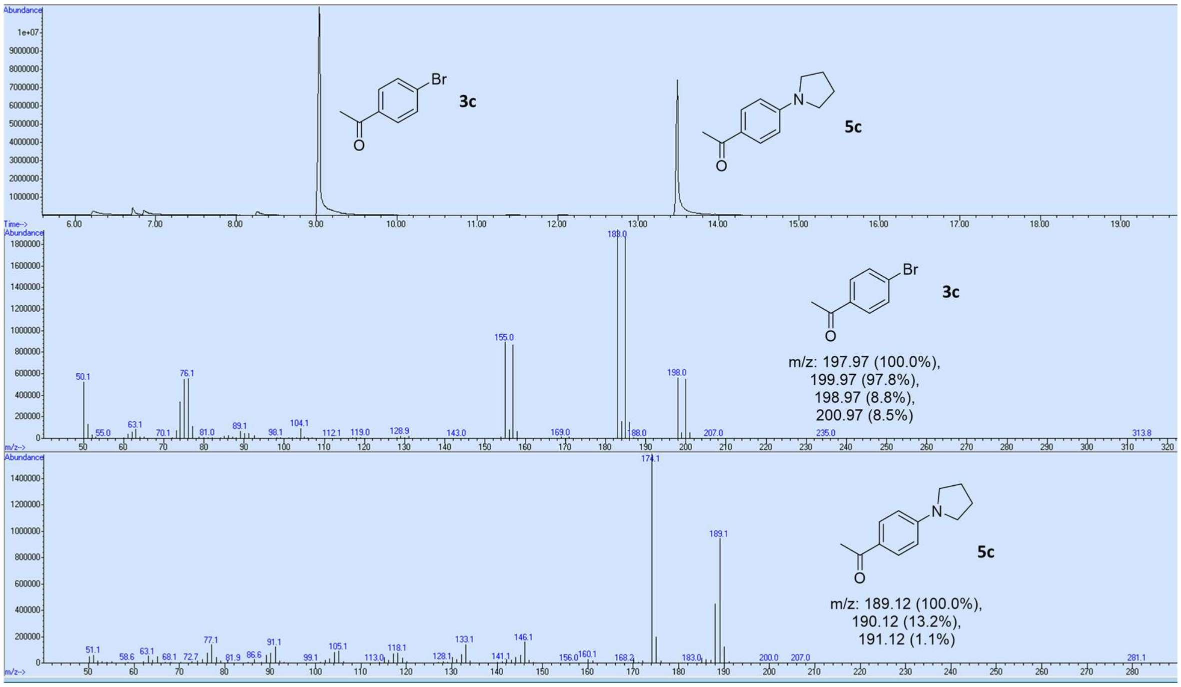

Supplementary Figure 44. Gas chromatogram of the reaction mixture and mass spectra of components using **3c** as the reagent. 2-bromobenzonitrile (0.05 mmol), PHT (12 mg), pyrrolidine (7.4 μL, 0.09 mmol), $NiBr_2$·glyme (0.8 mg, 0.0025 mmol), DABCO (12.3 mg, 0.11 mmol), N,N-dimethylacetamide (1 mL), Light 302 mW cm^{-2}, 168 h.

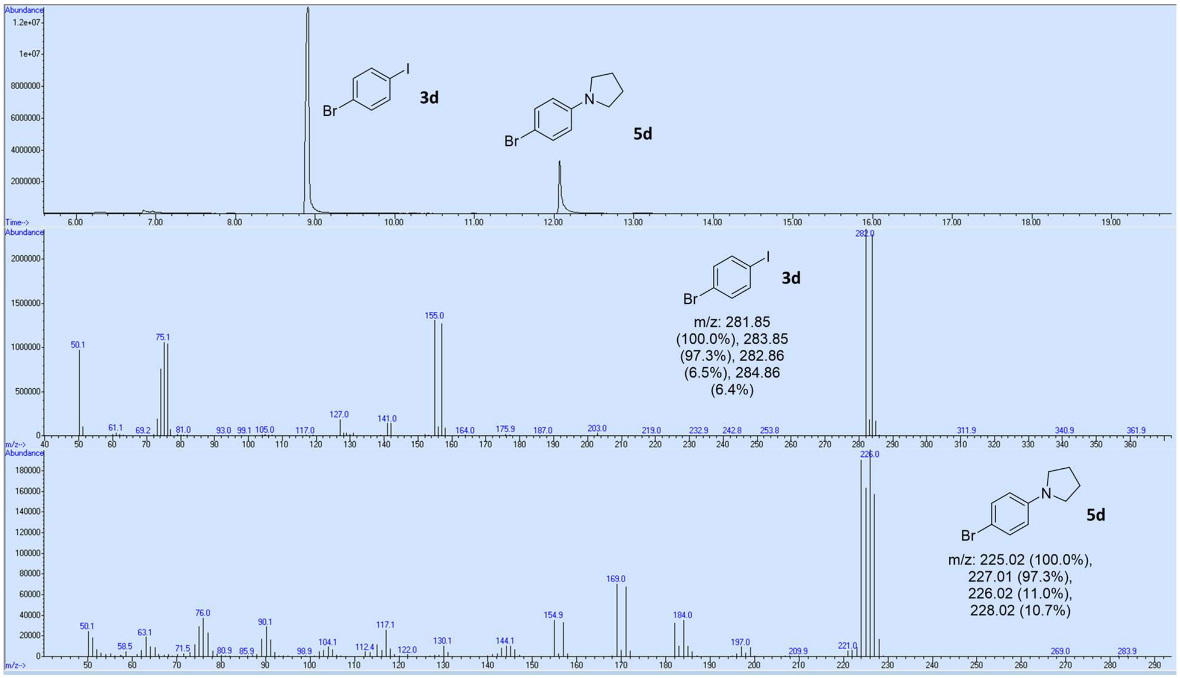

Supplementary Figure 45. Gas chromatogram of the reaction mixture and mass spectra of components using **3d** as the reagent. 1-Bromo-4-iodobenzene (0.05 mmol), PHT (12 mg), pyrrolidine (7.4 μL, 0.09 mmol), $NiBr_2$·glyme (0.8 mg, 0.0025 mmol), DABCO (12.3 mg, 0.11 mmol), N,N-dimethylacetamide (1 mL), Light 302 mW cm^{-2}, 168 h.

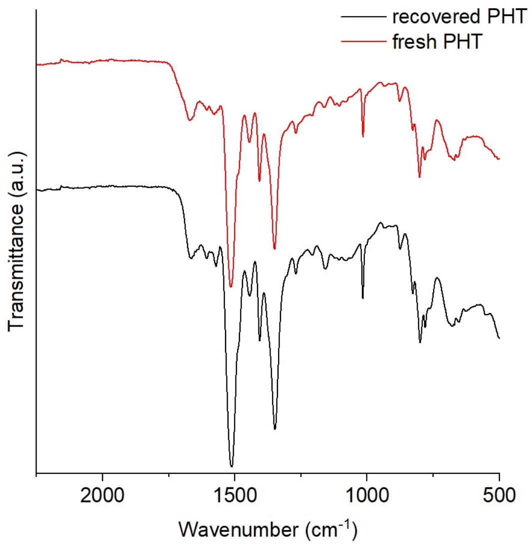

Supplementary Figure 46. FTIR spectra of fresh PHT and PHT recovered after dual Ni-photocatalytic C–N cross-coupling of pyrrolidine and 4-bromobenzonitrile.

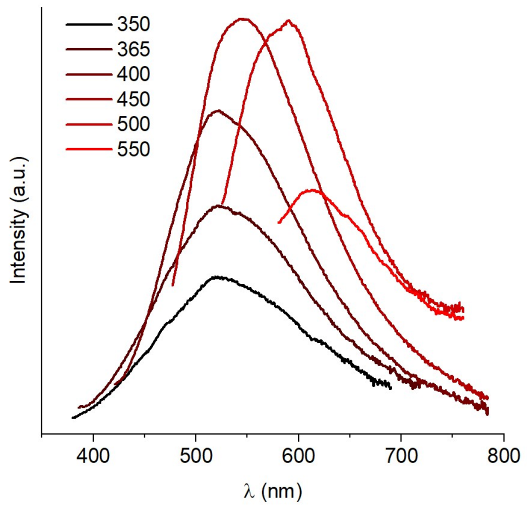

Supplementary Figure 47. PL spectra of PHT recovered after dual Ni-photocatalytic C–N cross-coupling of pyrrolidine and 4-bromobenzonitrile upon a range of λ_{exc}.

Supplementary Tables

Supplementary Table 1. Integrated area ratios of deconvoluted peaks in XPS spectra

PHT			
Element	Binding energy (eV)	Chemical state	Integrated area ratio (%)
C	284.8	C–C/C=C	82.5
	285.9	C=N	4.4
	287.0	C–O	11.3
	288.4	C–N	1.8
N	398.7	C=N–C	75.4
	400.0	NH_x	24.6
O	531.7	C=O	85.1
	533.1	C–OH	14.9
PYT			
Element	Binding energy (eV)	Chemical state	Integrated area ratio (%)
C	284.8	C–C/C=C	75.5
	286.0	C=N	6.0
	286.8	C–O	11.2
	288.8	C–N	7.2
N	398.7	C=N–C	78.1
	399.8	NH_x	21.9
O	532.1	C=O	79.3
	533.7	C–OH	20.7

Supplementary Table 2. Theoretical and measured elemental compositions of PHT and PYT.

Sample	Source	C (%)	N (%)	O (%)	H (%)
PHT	Theoretical	73.6	23.4	-	3.0
	SEM-EDX	78.7	18.9	2.4	-
	Elemental analysis	63.7	16.7	-	4.4
PYT	Theoretical	71.1	26.1	-	2.8
	SEM-EDX	74.6	21.1	4.1	74.6
	Elemental analysis	56.8	19.2	-	4.2

Supplementary Table 3. Fluorescence lifetime of PHT and PYT.

Sample	λ_{exc} (nm)	λ_{emi} (nm)	τ_1 (ns) (a_1)	τ_2 (ns) (a_2)	τ_3(ns) (a_3)	$\bar{\tau}$ (ns)[a]
PHT	375	400	0.007 (99.9%)	0.267 (0.08%)	5.36 (0.002%)	0.007
PHT	375	410	0.026 (97.5%)	0.340 (2.5%)	3.31 (0.08%)	0.036
PHT	375	420	0.056 (94.3%)	0.493 (5.4%)	2.86 (0.3%)	0.089
PHT	375	430	0.064 (91.7%)	0.520 (7.6%)	2.43 (0.7%)	0.115
PHT	375	440	0.119 (90.4%)	0.829 (9.0%)	3.92 (0.6%)	0.207
PHT	375	450	0.108 (86.8%)	0.739 (12.2%)	3.51 (1.0%)	0.220
PHT	375	460	0.157 (91.5%)	1.26 (4.4%)	2.10 (4.1%)	0.286
PHT	375	470	0.049 (85.3%)	0.45 (12.3%)	2.50 (2.4%)	0.158
PHT	375	480	0.122 (82.5%)	0.89 (15.5%)	3.67 (2.0%)	0.313
PHT	375	490	0.128 (80.4%)	0.968 (17.2%)	3.89 (2.4%)	0.361
PHT	375	500	0.136 (78.7%)	1.02 (18.5%)	3.97 (2.8%)	0.407
PHT	375	510	0.135 (77.7%)	1.03 (19.0%)	3.91 (3.3%)	0.430
PHT	375	520	0.135 (76.0%)	1.04 (20.3%)	4.01 (3.7%)	0.461
PHT	375	530	0.150 (74.8%)	1.11 (21.1%)	4.12 (4.1%)	0.515
PHT	375	540	0.143 (74.9%)	1.11 (20.9%)	4.16 (4.2%)	0.513
PHT	375	550	0.136 (74.4%)	1.06 (20.9%)	4.04 (4.7%)	0.511
PHT	375	560	0.135 (74.7%)	1.11 (21.0%)	4.24 (4.3%)	0.515
PHT	375	570	0.136 (74.9%)	1.07 (20.4%)	4.06 (4.7%)	0.511
PHT	375	580	0.094 (80.3%)	0.926 (15.8%)	3.70 (3.9%)	0.367
PHT	375	590	0.131 (75.8%)	1.05 (19.7%)	4.04 (4.5%)	0.489
PHT	375	600	0.131 (77.0%)	1.07 (18.7%)	4.03 (4.3%)	0.475
PHT	375	610	0.124 (77.4%)	1.01 (18.4%)	3.96 (4.2%)	0.449
PHT	375	620	0.120 (78.3%)	0.966 (17.2%)	3.75 (4.5%)	0.427
PHT	375	630	0.118 (80.0%)	1.01 (16.3%)	3.91 (3.7%)	0.403
PHT	375	640	0.112 (82.1%)	0.99 (14.6%)	3.86 (3.3%)	0.364

PHT	375	650	0.106 (83.7%)	1.00 (13.4%)	3.88 (2.9%)	0.334
PHT	375	660	0.109 (84.6%)	1.03 (12.8%)	3.93 (2.6%)	0.328
PHT	375	670	0.085 (86.6%)	0.86 (11.0%)	3.60 (2.4%)	0.253
PHT	375	680	0.086 (88.2%)	0.88 (9.8%)	3.64 (2.0%)	0.235
PHT	375	690	0.062 (90.6%)	0.74 (7.8%)	3.35 (1.6%)	0.167
PHT	375	700	0.055 (92.8%)	0.729 (6.0%)	3.30 (1.2%)	0.134
PHT	375	710	0.054 (94.5%)	0.77 (4.7%)	3.43 (0.8%)	0.116
PHT	375	720	0.037 (97.2%)	0.70 (2.4%)	3.18 (0.4%)	0.067
PHT	375	730	0.029 (98.5%)	0.69 (1.3%)	3.27 (0.2%)	0.045
PHT	375	740	0.043 (98.2%)	0.59 (1.6%)	3.05 (0.2%)	0.058
PYT	375	400	0.018 (99.8%)	0.315 (0.2%)	6.65 (0.02%)	0.020
PYT	375	410	0.022 (99.8%)	0.451 (0.1%)	6.57 (0.01%)	0.023
PYT	375	420	0.021 (99.7%)	0.355 (0.3%)	4.61 (0.02%)	0.022
PYT	375	430	0.032 (98.9%)	0.340 (1.0%)	3.00 (0.05%)	0.037
PYT	375	440	0.034 (97.3%)	0.282 (2.6%)	2.21 (0.1%)	0.043
PYT	375	450	0.049 (95.6%)	0.330 (4.2%)	2.10 (0.2%)	0.065
PYT	375	460	0.040 (96.5%)	0.325 (3.3%)	2.14 (0.2%)	0.053
PYT	375	470	0.072 (93.2%)	0.451 (6.4%)	2.58 (0.4%)	0.107
PYT	375	480	0.084 (91.5%)	0.503 (8.0%)	2.81 (0.5%)	0.132
PYT	375	490	0.082 (89.8%)	0.504 (9.4%)	2.63 (0.8%)	0.141
PYT	375	500	0.100 (87.9%)	0.570 (11.1%)	2.76 (1.0%)	0.18
PYT	375	510	0.104 (86.0%)	0.575 (12.7%)	2.85 (1.3%)	0.198
PYT	375	520	0.113 (84.2%)	0.607 (14.2%)	2.86 (1.6%)	0.227
PYT	375	530	0.115 (82.8%)	0.629 (15.4%)	2.95 (1.8%)	0.244
PYT	375	540	0.119 (81.1%)	0.617 (16.7%)	2.85 (2.2%)	0.263
PYT	375	550	0.125 (80.0%)	0.657 (17.7%)	3.02 (2.3%)	0.285
PYT	375	560	0.126 (80.4%)	0.694 (17.4%)	3.14 (2.2%)	0.290

PYT	375	570	0.132 (78.8%)	0.670 (18.4%)	2.98 (2.8%)	0.310
PYT	375	580	0.110 (79.6%)	0.604 (17.5%)	2.79 (2.9%)	0.274
PYT	375	590	0.131 (79.2%)	0.715 (18.2%)	3.22 (2.6%)	0.318
PYT	375	600	0.113 (78.5%)	0.630 (18.3%)	2.92 (3.2%)	0.296
PYT	375	610	0.147 (79.7%)	0.780 (17.6%)	3.34 (2.7%)	0.343
PYT	375	620	0.127 (79.2%)	0.695 (17.9%)	3.10 (2.9%)	0.316
PYT	375	630	0.121 (79.9%)	0.688 (17.4%)	3.12 (2.7%)	0.301
PYT	375	640	0.117 (81.0%)	0.674 (16.4%)	3.09 (2.6%)	0.286
PYT	375	650	0.110 (80.3%)	0.608 (16.6%)	2.82 (3.1%)	0.276
PYT	375	660	0.100 (81.4%)	0.593 (15.8%)	2.82 (2.8%)	0.255
PYT	375	670	0.094 (82.6%)	0.580 (14.8%)	2.75 (2.6%)	0.236
PYT	375	680	0.101 (83.4%)	0.609 (14.1%)	2.82 (2.5%)	0.239
PYT	375	690	0.087 (84.4%)	0.556 (13.2%)	2.72 (2.4%)	0.211
PYT	375	700	0.085 (86.3%)	0.560 (11.5%)	2.69 (2.1%)	0.194
PYT	375	710	0.067 (90.1%)	0.537 (8.5%)	2.63 (1.4%)	0.144
PYT	375	720	0.061 (92.8%)	0.548 (6.2%)	2.71 (1.0%)	0.118
PYT	375	730	0.057 (94.6%)	0.537 (4.6%)	2.59 (0.8%)	0.099
PYT	375	740	0.038 (97.0%)	0.442 (2.7%)	2.42 (0.3%)	0.057
PHT	470	515	0.081 (54.0%)	0.657 (38.0%)	3.80 (8.1%)	0.600
PHT	470	525	0.068 (65.6%)	0.831 (28.5%)	3.89 (5.9%)	0.512
PHT	470	535	0.092 (61.4%)	0.914 (30.8%)	3.80 (7.8%)	0.635
PHT	470	545	0.091 (49.4%)	0.965 (44.5%)	3.75 (6.1%)	0.624
PHT	470	555	0.115 (62.3%)	1.04 (28.7%)	3.80 (9.0%)	0.709
PHT	470	565	0.123 (61.5%)	1.04 (28.7%)	3.72 (9.8%)	0.738
PHT	470	575	0.100 (64.6%)	1.00 (26.6%)	3.73 (8.7%)	0.657
PHT	470	585	0.111 (63.1%)	1.01 (27.7%)	3.71 (9.2%)	0.691
PHT	470	595	0.092 (68.1%)	1.01 (24.1%)	3.76 (7.8%)	0.601

PHT	470	605	0.122 (63.2%)	1.04 (27.8%)	3.77 (9.0%)	0.708
PHT	470	615	0.139 (62.1%)	1.06 (28.3%)	3.75 (9.6%)	0.746
PHT	470	625	0.148 (62.2%)	1.07 (28.2%)	3.73 (9.6%)	0.753
PHT	470	635	0.101 (67.8%)	0.965 (24.5%)	3.69 (7.7%)	0.591
PHT	470	645	0.099 (68.4%)	0.938 (24.0%)	3.62 (7.6%)	0.567
PHT	470	655	0.108 (68.1%)	0.935 (23.9%)	3.53 (8.0%)	0.580
PHT	470	665	0.148 (64.9%)	1.03 (26.8%)	3.73 (8.3%)	0.681
PHT	470	675	0.128 (67.1%)	0.964 (25.2%)	3.61 (7.7%)	0.608
PHT	470	685	0.113 (69.2%)	0.911 (23.5%)	3.52 (7.2%)	0.548
PHT	470	695	0.109 (69.5%)	0.873 (23.6%)	3.49 (6.9%)	0.522
PHT	470	705	0.119 (69.8%)	0.887 (23.3%)	3.47 (6.9%)	0.530
PHT	470	715	0.104 (72.5%)	0.876 (21.6%)	3.50 (5.9%)	0.471
PHT	470	725	0.094 (73.9%)	0.808 (20.4%)	3.32 (5.7%)	0.424
PHT	470	735	0.079 (76.1%)	0.757 (19.0%)	3.34 (4.9%)	0.367
PHT	470	745	0.076 (77.2%)	0.730 (18.0%)	3.19 (4.8%)	0.345
PHT	470	755	0.077 (77.8%)	0.743 (17.6%)	3.20 (4.6%)	0.338
PHT	470	765	0.089 (76.4%)	0.752 (19.0%)	3.26 (4.6%)	0.362
PHT	470	775	0.076 (78.0%)	0.675 (17.4%)	3.00 (4.6%)	0.315
PHT	470	785	0.095 (75.2%)	0.712 (20.0%)	3.15 (4.8%)	0.364
PHT	470	795	0.095 (76.3%)	0.727 (19.4%)	3.22 (4.3%)	0.352
PHT	470	805	0.101 (75.0%)	0.711 (20.4%)	3.14 (4.6%)	0.365
PHT	470	815	0.117 (72.9%)	0.708 (22.2%)	3.15 (4.9%)	0.397
PHT	470	825	0.105 (75.5%)	0.706 (20.2%)	3.13 (4.3%)	0.356
PHT	470	835	0.107 (74.9%)	0.680 (20.6%)	3.11 (4.5%)	0.360
PHT	470	845	0.097 (76.7%)	0.662 (19.2%)	3.03 (4.1%)	0.325
PHT	470	855	0.103 (77.7%)	0.695 (18.6%)	3.19 (3.7%)	0.328
PHT	470	865	0.106 (77.3%)	0.670 (18.6%)	3.01 (4.1%)	0.330

PYT	470	525	0.290 (84.1%)	1.19 (13.3%)	4.64 (2.6%)	0.523
PYT	470	535	0.209 (71.7%)	0.990 (25.6%)	3.78 (2.7%)	0.507
PYT	470	545	0.216 (70.1%)	0.992 (26.7%)	3.65 (3.2%)	0.533
PYT	470	555	0.182 (64.8%)	0.835 (30.0%)	3.19 (5.2%)	0.534
PYT	470	565	0.188 (65.6%)	0.880 (29.3%)	3.28 (5.1%)	0.549
PYT	470	575	0.190 (65.5%)	0.900 (29.2%)	3.32 (5.2%)	0.561
PYT	470	585	0.176 (63.6%)	0.859 (30.3%)	3.24 (6.1%)	0.571
PYT	470	595	0.193 (63.9%)	0.910 (30.0%)	3.34 (6.1%)	0.600
PYT	470	605	0.181 (63.0%)	0.870 (30.3%)	3.27 (6.6%)	0.595
PYT	470	615	0.153 (61.0%)	0.773 (31.4%)	3.14 (7.6%)	0.575
PYT	470	625	0.174 (60.6%)	0.820 (31.6%)	3.16 (7.8%)	0.612
PYT	470	635	0.159 (61.1%)	0.794 (31.1%)	3.18 (7.8%)	0.593
PYT	470	645	0.183 (63.2%)	0.878 (29.6%)	3.21 (7.2%)	0.607
PYT	470	655	0.201 (64.3%)	0.930 (28.6%)	3.27 (7.1%)	0.627
PYT	470	665	0.160 (61.7%)	0.799 (30.4%)	3.08 (7.9%)	0.584
PYT	470	675	0.150 (61.3%)	0.761 (30.5%)	3.02 (8.2%)	0.573
PYT	470	685	0.148 (59.8%)	0.745 (31.6%)	2.94 (8.6%)	0.577
PYT	470	695	0.146 (60.2%)	0.762 (31.4%)	2.94 (8.4%)	0.574
PYT	470	705	0.170 (61.0%)	0.850 (30.9%)	2.97 (8.1%)	0.605
PYT	470	715	0.156 (60.2%)	0.783 (30.9%)	2.84 (8.9%)	0.586
PYT	470	725	0.155 (60.6%)	0.784 (31.0%)	2.80 (8.4%)	0.573
PYT	470	735	0.170 (61.3%)	0.796 (30.6%)	2.82 (8.1%)	0.577
PYT	470	745	0.142 (61.6%)	0.738 (30.8%)	2.74 (7.6%)	0.523
PYT	470	755	0.143 (63.5%)	0.730 (29.0%)	2.72 (7.5%)	0.505
PYT	470	765	0.130 (64.7%)	0.701 (28.3%)	2.72 (7.0%)	0.473
PYT	470	775	0.134 (66.0%)	0.728 (27.4%)	2.72 (6.6%)	0.468
PYT	470	785	0.117 (66.8%)	0.675 (26.8%)	2.67 (6.4%)	0.431

PYT	470	795	0.111 (68.5%)	0.668 (25.4%)	2.68 (6.1%)	0.409
PYT	470	805	0.114 (69.2%)	0.670 (24.9%)	2.66 (5.9%)	0.402
PYT	470	815	0.109 (70.1%)	0.659 (24.4%)	2.62 (5.5%)	0.381
PYT	470	825	0.100 (72.3%)	0.658 (22.7%)	2.58 (5.0%)	0.351
PYT	470	835	0.103 (72.3%)	0.638 (22.7%)	2.58 (5.0%)	0.349
PYT	470	845	0.093 (73.8%)	0.599 (21.5%)	2.58 (4.7%)	0.320
PYT	470	855	0.081 (76.8%)	0.580 (19.1%)	2.54 (4.1%)	0.277
PYT	470	865	0.082 (76.4%)	0.571 (19.5%)	2.59 (4.1%)	0.280
PHT	640	695	0.645 (59.7%)	0.160 (29.2%)	3.33 (11.1%)	0.801
PHT	640	705	0.620 (62.9%)	0.176 (24.6%)	3.17 (12.5%)	0.830
PHT	640	715	0.671 (47.4%)	0.124 (43.3%)	3.24 (9.2%)	0.670
PHT	640	725	0.195 (49.5%)	0.786 (41.3%)	3.30 (9.2%)	0.725
PHT	640	735	0.197 (53.6%)	0.832 (37.7%)	3.28 (8.6%)	0.704
PHT	640	745	0.252 (64.0%)	1.08 (28.9%)	3.48 (7.0%)	0.719
PHT	640	755	0.200 (60.5%)	0.866 (32.3%)	3.23 (7.2%)	0.634
PHT	640	765	0.195 (61.7%)	0.846 (31.2%)	3.12 (7.1%)	0.606
PHT	640	775	0.198 (63.8%)	0.875 (29.7%)	3.17 (6.5%)	0.591
PHT	640	785	0.208 (66.3%)	0.899 (27.9%)	3.20 (5.8%)	0.575
PHT	640	795	0.183 (64.3%)	0.779 (29.8%)	3.02 (5.9%)	0.528
PHT	640	805	0.231 (65.4%)	0.813 (28.8%)	3.09 (5.8%)	0.564
PHT	640	815	0.232 (65.1%)	0.754 (28.9%)	2.93 (6.0%)	0.544
PHT	640	825	0.268 (69.5%)	0.846 (25.3%)	3.07 (5.2%)	0.560
PHT	640	835	0.258 (68.4%)	0.809 (26.6%)	3.01 (5.0%)	0.541
PHT	640	845	0.248 (68.6%)	0.751 (26.5%)	2.93 (4.9%)	0.514
PHT	640	855	0.240 (70.4%)	0.755 (25.1%)	2.95 (4.5%)	0.490
PHT	640	865	0.230 (71.7%)	0.732 (24.1%)	2.92 (4.2%)	0.463
PHT	640	875	0.146 (70.5%)	0.633 (26.4%)	2.99 (3.1%)	0.362

PHT	640	885	0.136 (77.2%)	0.687 (20.2%)	2.98 (2.6%)	0.322
PYT	640	695	0.269 (43.5%)	1.11 (42.4%)	2.78 (14.1%)	0.981
PYT	640	705	1.10 (45.1%)	0.237 (41.5%)	2.70 (13.4%)	0.956
PYT	640	715	1.04 (46.1%)	0.214 (40.7%)	2.59 (13.2%)	0.907
PYT	640	725	1.05 (44.7%)	0.217 (43.9%)	2.57 (11.4%)	0.857
PYT	640	735	0.219 (47.0%)	1.06 (43.2%)	2.59 (9.8%)	0.815
PYT	640	745	0.203 (49.3%)	1.01 (40.9%)	2.54 (9.8%)	0.761
PYT	640	755	0.180 (50.9%)	0.970 (39.6%)	2.52 (9.5%)	0.714
PYT	640	765	0.198 (52.8%)	1.01 (38.2%)	2.56 (9.0%)	0.721
PYT	640	775	0.177 (55.5%)	0.999 (35.8%)	2.52 (8.6%)	0.674
PYT	640	785	0.190 (55.9%)	1.011 (35.3%)	2.48 (8.8%)	0.682
PYT	640	795	0.158 (56.4%)	0.925 (34.0%)	2.35 (9.6%)	0.629
PYT	640	805	0.142 (58.6%)	0.896 (32.7%)	2.32 (8.7%)	0.579
PYT	640	815	0.140 (61.1%)	0.898 (30.6%)	2.26 (8.3%)	0.549
PYT	640	825	0.148 (62.1%)	0.915 (30.6%)	2.33 (7.3%)	0.542
PYT	640	835	0.113 (67.1%)	0.849 (26.1%)	2.23 (6.8%)	0.449
PYT	640	845	0.118 (67.0%)	0.817 (26.0%)	2.21 (7.0%)	0.445
PYT	640	855	0.106 (69.9%)	0.798 (23.8%)	2.21 (6.3%)	0.403
PYT	640	865	0.097 (71.9%)	0.758 (21.9%)	2.15 (6.2%)	0.369
PYT	640	875	0.098 (72.5%)	0.753 (21.2%)	2.12 (6.3%)	0.364
PYT	640	885	0.093 (72.7%)	0.719 (20.8%)	2.05 (6.5%)	0.351
PYT	640	895	0.086 (74.3%)	0.671 (19.1%)	1.97 (6.6%)	0.322

[a] Amplitude average lifetime.

Supplementary Table 4. Influence of explicitly added quantities of H_2O_2 on the yield of 4-bromoanisole.

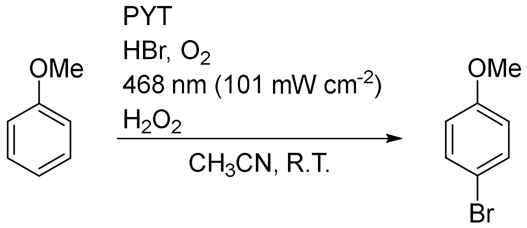

Entry	H_2O_2	Yield (%)	Conversion (%)
1	0.6 µL (0.01 eq.)	85	100
2	3 µL (0.05 eq.)	89	100
3	12 µL (0.2 eq.)	79	100
4	30 µL (0.5 eq.)	72	100
5	60 µL (1 eq.)	75	100
6	120 µL (2 eq.)	77	100

Reaction conditions: anisole (65 µL, 0.6 mmol), PYT (4 mg), HBr (0.6 mL, 6 mmol, 48 wt. %), 468 nm (101 mW cm^{-2}), 48 h, MeCN (3 mL), O_2 (1 bar).

Supplementary Table 5. Combustion elemental analysis of fresh PYT and recovered after the photocatalytic oxidative bromination of anisole.

Entry	C (%)	N(%)	H (%)
PYT	56.8	19.2	4.2
PYT recycled	55.1±1	17.0±0.5	4.1±0.2

Supplementary Table 6. Control experiments for leaching of photocatalytically active organic moieties from PYT.

Entry	Yield (%)	Conversion (%)
1[a]	99	100
2[b]	0	0

[a] Reaction conditions: anisole (0.6 mmol); HBr (0.6 mL, 48 wt. %); MeCN (3 mL); PYT (4 mg); electron scavenger – O_2; at room temperature; 48 h; 461 nm (101 mW cm^{-2}).

[b] After the photocatalytic experiment performed according to the conditions specified in entry 1, PYT was separated from the reaction mixture by centrifugation at 13000 rpm. The solution was loaded into the clean photoreactor. A new portion of anisole (0.6 mmol) and HBr (0.6 mL, 48 wt. %) was added to the solution. The solution was stirred under irradiation with blue LED (461 nm, 101 mW cm^{-2}) for 48 h under atmosphere of O_2.

Supplementary Table 7. Photocatalytic oxidative bromination with PYT versus oxidative bromination of electron rich aromatic compounds using a mixture of H_2O_2 and HBr in dark.

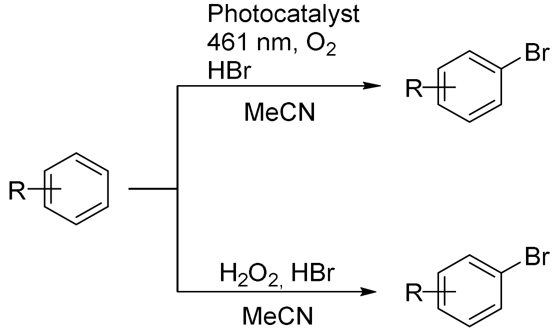

Photocatalytic oxidative bromination with PYT[a]					Oxidative bromination in dark[b]	
Entry	Substrate	Product	Time, h	Yield, %	Product	Yield (Conversion), %[e]
1	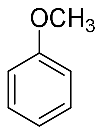	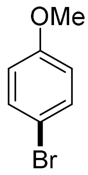	48	97[c]	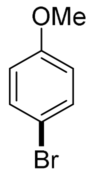	89(100)
2	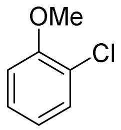	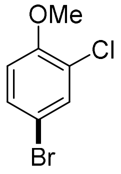	72	80[c]	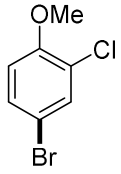	75(75)
3	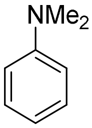	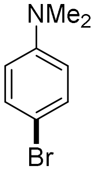 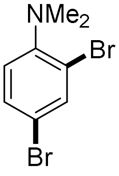	72	85 (4:1)[d]	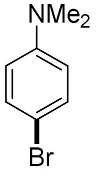	73(73)
4	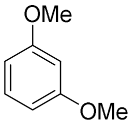	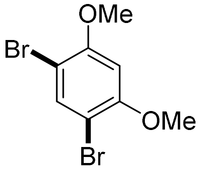	48	95[c]	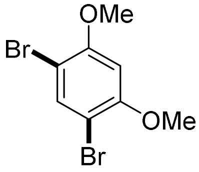	74(74)
5	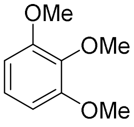	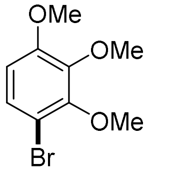	24	75[c]	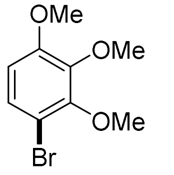	69(69)
6	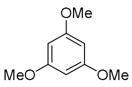	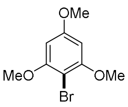 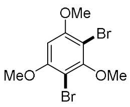	24	81 (1:1)[c]	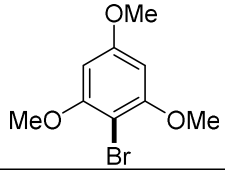	74(74)

[a] Reaction conditions: substrate (0.6 mmol), HBr (0.6 mL, 48 wt. %), MeCN (3 mL), PYT (4 mg), electron scavenger – O_2, blue LED module 2 (461 nm, 101 mW cm^{-2}) for entry 1, 3-5 and white LED module (400-760 nm, 203 mW cm^{-2}) for entry 2, at room temperature.

[b] Reaction conditions: substrate (0.6 mmol), HBr (0.6 mL, 48 wt. %), H_2O_2 (10.6 mmol, 1.08 mL, 30 wt. %), MeCN (3 mL), at room temperature.

[c] Isolated yields.

[d] Yields determined by ^1H NMR with 1,4-dinitrobenzene as internal standard.

[e] Yield and conversion (in parentheses) determined via GC-MS.

Supplementary Table 8. Oxidative photocatalytic bromination of 1,3,5-trimethoxybenzene with KBr.[a]

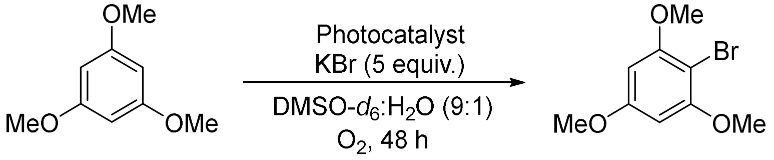

Entry[a]	Catalyst	Light	Yield (%)[b]	Conversion (%)[b]
1	PYT 12 mg	400 nm	85	100
2	PHT 12 mg	400 nm	83	100
3	PYT 12 mg	465 nm	52	88
4	PHT 12 mg	465 nm	14	14

[a] Reaction conditions: anisole (21.7 µL, 0.2 mmol), KBr (119 mg, 1 mmol), 48 h, DMSO-d_6:H_2O (1 mL, 9:1), O_2 (1 bar). Condition were adopted from the reference [5]. Yield and conversion were determined by ^{1}H NMR with internal standard.

Supplementary Table 9. Photocatalytic oxidative bromination of 3,4-ethylenedioxythiophene with PYT using KBr as bromine source.

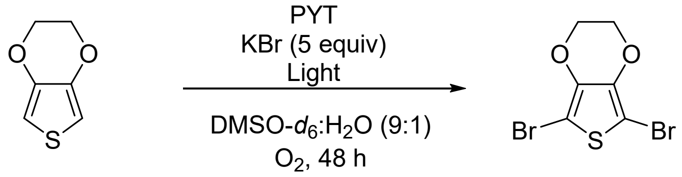

**Entry**[a]	**Light**	**Yield (%)**[b]	**Conversion (%)**[b]
1	465 nm	44	100
2	400 nm	42	100

[a] Reaction conditions: PYT (12 mg), 3,4-ethylenedioxythiophene (21.4 μL, 0.2 mmol), KBr (119 mg, 1 mmol), 48 h, DMSO-d_6:H_2O (1 mL, 9:1), O_2 (1 bar). Condition were adopted from the reference [5]. Yield and conversion were determined by ^1H NMR with internal standard.

Supplementary Table 10. Role of acid in photocatalytic oxidative halogenation of electron rich aromatic compounds.

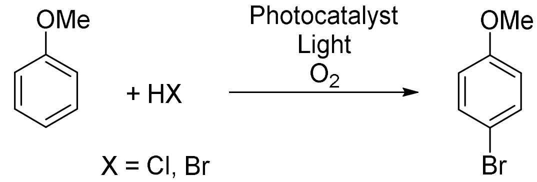

Entry	Photocatalyst	Role of acid	Reference
1	Microporous organic polymers (heterogeneous)	Participates in PCET to generate $HO_2^{\bullet}$ from O_2.	1
2	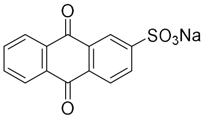 sodium anthraquinone-2-sulfonate (SAS, homogeneous)	Protonation of the photocatalyst increases excited oxidation potential of the photocatalyst, which in turn enables oxidation of thermodynamically more stable substrates via PCET. SAS-H• as the intermediate in the photocatalytic cycle is postulated.	14
3	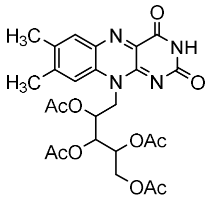 Riboflavin tetraacetate (RFT) (homogeneous)	Acetic acid is converted into peracetic acid in situ, which in turn enables oxidation of Cl^{-} to OCl^{-}.	15
4	Potassium poly(heptazine imide)	Participates in PCET to generate H_2O_2 from O_2.	16
5	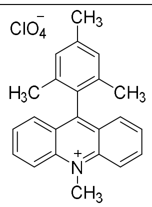 $[Acr\text{-}Mes]^{+}ClO_4^{-}$	Participates in PCET to generate $HO_2^{\bullet}$ from O_2.	17

Supplementary Table 11. An attempt to enable photocatalytic oxidative bromination of anisole with PYT using KBr as bromine source.[a]

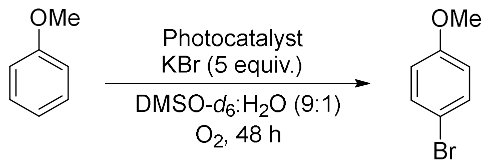

Entry	Photocatalyst	Light	Yield (%)	Conversion (%)
1	PYT 12 mg	400 nm	0	0
2	PHT 12 mg	400 nm	0	0
3	PYT 12 mg	465 nm	0	0
4	PHT 12 mg	465 nm	0	0

[a] Reaction conditions: anisole (21.7 µL, 0.2 mmol), KBr (119 mg, 1 mmol), 48 h, DMSO-d_6:H_2O (1 mL, 9:1), O_2 (1 bar). Condition were adopted from the reference [5]. Yield and conversion were determined by ^{1}H NMR with internal standard.

Supplementary Table 12. Combustion elemental analysis of fresh PHT and recovered after dual Ni-photocatalytic C–N cross-coupling.

Entry	C (%)	N(%)	H (%)
Fresh PHT	63.7	16.7	4.4
Recovered PHT	62.1±0.02	16.1±0.04	4.1±0.03

Supplementary References

1. Li R*, et al.* Photocatalytic selective bromination of electron-rich aromatic compounds using microporous organic polymers with visible light. *ACS Catal* **6**, 1113-1121 (2016).

2. Ye C*, et al.* Enhanced Driving Force and Charge Separation Efficiency of Protonated g-C3N4 for Photocatalytic O2 Evolution. *ACS Catal* **5**, 6973-6979 (2015).

3. Valdez CN, Schimpf AM, Gamelin DR, Mayer JM. Proton-Controlled Reduction of ZnO Nanocrystals: Effects of Molecular Reductants, Cations, and Thermodynamic Limitations. *J Am Chem Soc* **138**, 1377-1385 (2016).

4. Roth HG, Romero NA, Nicewicz DA. Experimental and Calculated Electrochemical Potentials of Common Organic Molecules for Applications to Single-Electron Redox Chemistry. *Synlett* **27**, 714-723 (2016).

5. Ghosh I, Khamrai J, Savateev A, Shlapakov N, Antonietti M, Konig B. Organic semiconductor photocatalyst can bifunctionalize arenes and heteroarenes. *Science* **365**, 360-366 (2019).

6. Corcoran EB*, et al.* Aryl amination using ligand-free Ni(II) salts and photoredox catalysis. *Science* **353**, 279-283 (2016).

7. Ghosh I, Ghosh T, Bardagi JI, König B. Reduction of aryl halides by consecutive visible light-induced electron transfer processes. *Science* **346**, 725-728 (2014).

8. Ghosh I, Konig B. Chromoselective photocatalysis: controlled bond activation through light-color regulation of redox potentials. *Angew Chem Int Ed* **55**, 7676-7679 (2016).

9. Marin M, Miranda MA, Marin ML. A comprehensive mechanistic study on the visible-light photocatalytic reductive dehalogenation of haloaromatics mediated by $Ru(bpy)_3Cl_2$. *Catal Sci Technol* **7**, 4852-4858 (2017).

10. Weinberg NL, Weinberg HR. Electrochemical oxidation of organic compounds. *Chem Rev* **68**, 449-523 (1968).

11. Savateev A*, et al.* Potassium poly(heptazine imide): transition metal-free solid-state triplet sensitizer in cascade energy transfer and [3+2]-cycloadditions. *Angew Chem Int Ed* **59**, 15061-15068 (2020).

12. Pavlishchuk VV, Addison AW. Conversion constants for redox potentials measured versus different reference electrodes in acetonitrile solutions at 25°C. *Inorg Chim Acta* **298**, 97-102 (2000).

13. Lowry MS*, et al.* Single-Layer Electroluminescent Devices and Photoinduced Hydrogen Production from an Ionic Iridium(III) Complex. *Chem Mater* **17**, 5712-5719 (2005).

14. Petzold D, König B. Photocatalytic Oxidative Bromination of Electron-Rich Arenes and Heteroarenes by Anthraquinone. *Adv Synth Catal* **360**, 626-630 (2018).

15. Hering T, Mühldorf B, Wolf R, König B. Halogenase-Inspired Oxidative Chlorination Using Flavin Photocatalysis. *Angew Chem Int Ed* **55**, 5342-5345 (2016).

16. Markushyna Y*, et al.* Halogenation of aromatic hydrocarbons by halide anion oxidation with poly(heptazine imide) photocatalyst. *Appl Catal B* **248**, 211-217 (2019).

17. Ohkubo K, Mizushima K, Iwata R, Fukuzumi S. Selective photocatalytic aerobic bromination with hydrogen bromidevia an electron-transfer state of 9-mesityl-10-methylacridinium ion. *Chem Sci* **2**, 715-722 (2011).
